# Staphylococcal *saoABC* Operon Codes for a DNA-Binding Protein SaoC Implicated in the Response to Nutrient Deficit

**DOI:** 10.3390/ijms23126443

**Published:** 2022-06-09

**Authors:** Michal Bukowski, Maja Kosecka-Strojek, Anna Madry, Rafal Zagorski-Przybylo, Tomasz Zadlo, Katarzyna Gawron, Benedykt Wladyka

**Affiliations:** 1 Department of Analytical Biochemistry, Faculty of Biochemistry, Biophysics and Biotechnology, Jagiellonian University in Krakow, 30-387 Krakow, Poland; anna.madry@doctoral.uj.edu.pl (A.M.); raphael.z.p@gmail.com (R.Z.-P.); tomasz.zadlo@doctoral.uj.edu.pl (T.Z.); benedykt.wladyka@uj.edu.pl (B.W.); 2 Department of Microbiology, Faculty of Biochemistry, Biophysics and Biotechnology, Jagiellonian University in Krakow, 30-387 Krakow, Poland; maja.kosecka-strojek@uj.edu.pl; 3 Department of Molecular Biology and Genetics, Faculty of Medical Sciences in Katowice, Medical University of Silesia, 40-752 Katowice, Poland; kgawron@sum.edu.pl

**Keywords:** *Staphylococcus*, *Staphylococcus aureus*, transcription factors, regulation of gene expression, stress response, virulence

## Abstract

Whilst a large number of regulatory mechanisms for gene expression have been characterised to date, transcription regulation in bacteria still remains an open subject. In clinically relevant and opportunistic pathogens, such as *Staphylococcus aureus*, transcription regulation is of great importance for host-pathogen interactions. In our study we investigated an operon, exclusive to staphylococci, that we name *saoABC*. We showed that SaoC binds to a conserved sequence motif present upstream of the *saoC* gene, which likely provides a negative feedback loop. We have also demonstrated that *S.* *aureus* Δ*saoB* and Δ*saoC* mutants display altered growth dynamics in non-optimal media; Δ*saoC* exhibits decreased intracellular survival in human dermal fibroblasts, whereas Δ*saoB* produces an elevated number of persisters, which is also elicited by inducible production of SaoC in Δ*saoB*Δ*saoC* double mutant. Moreover, we have observed changes in the expression of *saoABC* operon genes during either depletion of the preferential carbon or the amino acid source as well as during acidification. Comparative RNA-Seq of the wild type and Δ*saoC* mutant demonstrated that SaoC influences transcription of genes involved in amino acid transport and metabolism, and notably of those coding for virulence factors. Our results suggest compellingly that *saoABC* operon codes for a DNA-binding protein SaoC, a novel staphylococcal transcription factor, and its antagonist SaoB. We linked SaoC to the response to nutrient deficiency, a stress that has a great impact on host-pathogen interactions. That impact manifests in SaoC influence on persister formation and survival during internalisation to host cells, as well as on the expression of genes of virulence factors that may potentially result in profound alternations in the pathogenic phenotype. Investigation of such novel regulatory mechanisms is crucial for our understanding of the dynamics of interactions between pathogenic bacteria and host cells, particularly in the case of clinically relevant, opportunistic pathogens such as *Staphylococcus aureus*.

## 1. Introduction

Regulation of gene transcription in bacteria is realised in many ways. Beginning with simple transcription factors (TFs) that are proteins composed of a signal-sensing and an effector domain [1], moving through to two-component systems, which are considered to be the most numerous bacterial regulatory systems [2], and arriving at alternative sigma factors of the bacterial RNA polymerase (RNAP), each recognising its own promoter sequences [3]. All the aforementioned mechanisms rely on proteins as the ultimate components. However, it shall not be overlooked that to a lesser extent transcriptional regulation in bacteria involves small RNA molecules as well, with 6S RNA being the most obvious example [4]. Regulation of gene expression at the transcriptional level plays a crucial role in bacteria by seemingly being the most important way of providing necessary plasticity for interactions between bacterial cells and their environment. In the case of pathogenic species, it translates into orchestrating the successful colonisation of the host, progression of an infection, its spread from the initial colonisation site as well as biofilm and persister formation [5,6,7,8,9,10]. A plethora of diverse bacterial regulatory systems have been characterised so far. Nonetheless bacterial genomes contain a considerable number of what is known today merely as hypothetical coding sequences, and there are certainly among them such coding for transcription regulation mechanisms that are yet to be characterised. Recent reports suggest that *Staphylococcus aureus*, a human opportunistic pathogen of clinical importance, is no exception [11,12,13,14].

Up until now, three alternative RNAP sigma factors (B, H, S), 16 two-component systems and a much higher number of simple transcription factors have been described for *S. aureus* [15]. Advances in high-throughput techniques enable relatively quick, albeit detailed, regulon analysis by whole transcriptome sequencing, which facilitates research on yet uncharacterised TFs. Reports on such two newly characterised transcription factors from *S. aureus* have been released quite recently. Yoe et al. [12] investigated MbtS that is degraded by AAA+ protease FtsH, and, as the authors postulate, the first membrane-bound transcription factor (MTF) described for Gram-positive bacteria. The gene that encodes MbtS is preceded by two other genes, and the operon seems to be controlled by two promoters: one upstream of the whole operon, and one upstream of *mbtS*. Although MbtS still requires in-depth investigation, it has been linked to the virulence of *S. aureus* in some settings. Disruption of *mbtS* leads to larger skin lesions in a murine model of skin infection; however, the disruption has apparently no effect when compared to the wild type pathogen upon intravenous administration in mice. Interestingly, the authors did not observe any significant changes in the transcriptome in *mbtS* mutant when grown in optimal conditions [12]. Another study, recently described by Groma et al. [11], showed that staphylococcal transcription factor LTTR was also demonstrated to influence virulence of *S. aureus* by providing efficient secondary organ colonisation during blood infection. Induction of LTTR elevates transcription of genes engaged in branched-chain amino acid biosynthesis of a methionine sulfoxide reductase as well as a copper transporter, and decreases transcription of genes coding for urease and those involved in biosynthesis of pyrimidine nucleotides. Next to MbtS and LTTR, which have been investigated relatively recently, there are several other transcription factors of *S. aureus* that are similar to the aforementioned, such as CcpA [16,17,18], PurR [13,19,20] or CodY [14,21,22]. All of these have one thing in common, namely constituting a link between the metabolic state of a bacterial cell and the virulent phenotype, as one is interwoven with the other to a great extent [23]. Currently, transcription regulation is also the subject of extensive investigation in the context of biofilm and persister formation. These, respectively, are complex multicellular structures and metabolic phenotype, and both facilitate survival of bacteria in adverse conditions. Research in this area yet again explores possible links between the metabolic state of a bacterial cell and its phenotype in the context of pathogenesis [24], and points at certain transcription regulatory systems linked to this phenomenon [25].

In this study we undertook an investigation into a yet uncharacterised staphylococcal operon that we name *saoABC* (stress-associated operon) [26]. One of its 3 open reading frames codes for a DNA-binding protein SaoC, which, as we argue, is a transcription factor. Our interest was drawn to *saoC* when we found out that its transcript is statistically under-represented with UAUU sequences recognised by PemK-Sa1 ribonuclease of a class II toxin–antitoxin system [27], which suggested the possible importance of *saoC* for the stress response and virulence in *S. aureus* [28]. Although the role of the products of two other *saoABC* operon open reading frames is elusive, we argue that SaoB is highly likely an antagonist of SaoC. According to our findings SaoC appears to be implicated in the response to nutrient deficiency, a stress that has great impact on host-pathogen interactions. However, SaoC impact on staphylococcal metabolism extends over persister formation, internalisation of bacteria to host cells, as well as the expression of virulence factors. Moreover, SaoC is potentially a candidate for another example, rare among Gram-positive bacteria, of a membrane-bound transcription factor.

## 2. Results

**Figure 1:**
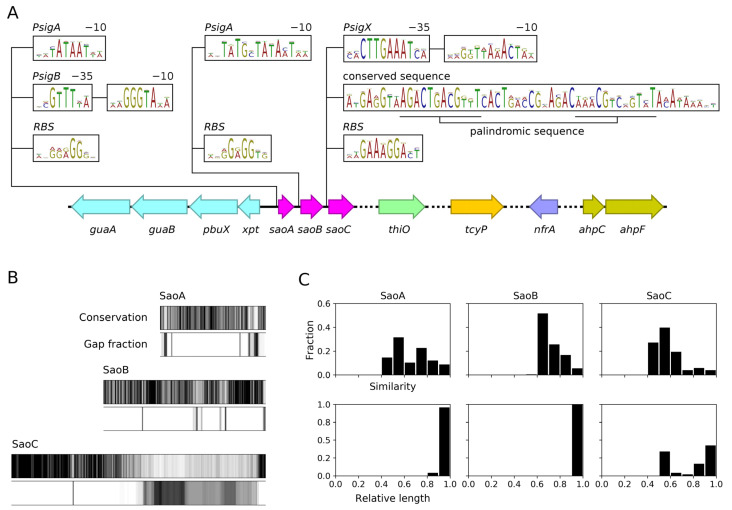
The genetic context of *saoABC* operon and variability of sequences of the encoded proteins among 47 staphylococcal species. (**A**) Upstream of *saoABC* operon there is an operon coding for xanthine phosphoribosyltransferase (*xpt*), purine permease (*pbuX*), IMP dehydrogenase (*guaB*) and glutamine-hydrolyzing GMP synthase (*guaA*), genes coding for proteins related to de novo guanine biosynthesis and the purine salvage pathway. Downstream, genes coding for glycine oxidase (*thiO*), L-cystine transporter (*tcyP*), oxygen-insensitive NADPH nitroreductase (*nfrA*) as well as an operon coding for alkyl hydroperoxide reductase subunits C (peroxiredoxin, *ahpC*) and F (*ahpF*). Dashed lines represent variable sequence fragments where other genes are found on a species-specific basis. Upstream of *saoABC* operon genes a few conserved motifs are found. Next to promoters for σ^A^ (*PsigA*) and σ^B^ (*PsigB*) as well as ribosome binding sites (RBS) unknown to date in staphylococci motifs are observed. These include a putative promoter for an unknown putative σ^X^ factor (*PsigX*) and a conserved span of sequence containing a palindromic repeat. (**B**) Conservation plots of SaoA, SaoB and SaoC protein sequences show that whereas SaoA and SaoB protein sequences are conserved among different staphylococcal species, SaoC sequence displays an unusual pattern of conservation with approximately 150 aa N-terminal fragment and a short 12 aa C-terminal motif being conserved, and the remaining span of the sequence variable in respect to amino acid sequence as well as its length. (**C**) Pairwise sequence similarity among SaoA, SaoB and SaoC protein sequences (top) and their relative length (bottom). SaoA and SaoB sequences are conserved and of very similar lengths among all analysed staphylococcal species with SaoB sequence being the most conserved of all three. On the other hand, SaoC sequence is relatively less conserved and of highly variable length with discernible short and long variants.

**The *saoABC* operon is prevalent among and exclusive to staphylococci and displays unique sequence characteristics**. Knowing that *saoC* transcript possibly avoids endoribonucleolytic activity of PemK-Sa1 toxin, which suggested that it could potentially be implicated in the stress response [28], and also knowing from a preliminary analysis that the gene codes for a hypothetical and likely DNA-binding protein, we decided to undertake an investigation into the function of SaoC. We started the research with a thorough analysis of the genetic neighbourhood of *saoC* and the search for sequences coding for homologous proteins in other bacteria. That led to the identification of *saoABC* operon, which codes for three hypothetical proteins (SaoA, SaoB and SaoC), and is present exclusively in bacteria of the *Staphylococcus* genus. The analysis of whole genome sequencing (WGS) results available in the GenBank database for 51 different staphylococcal species allowed us to trace the operon’s presence to 47 of them (Supplementary List S1). Detailed comparative analysis of sequences derived from those species revealed that in the closest, most conserved neighbourhood of *saoABC* operon when moving upstream there are: an operon of four genes coding for xanthine phosphoribosyltransferase (*xpt*), purine permease (*pbuX*), IMP dehydrogenase (*guaB*) and glutamine-hydrolyzing GMP synthase (*guaA*), all found in 46 species; when moving downstream: a gene coding for glycine oxidase (*thiO*) found in 13 species, L-cystine transporter (*tcyP*) and oxygen-insensitive NADPH nitroreductase (*nfrA*) both found in 44 species, as well as an operon coding for alkyl hydroperoxide reductase subunits C (peroxiredoxin, *ahpC*) and F (*ahpF*) found in 43 species. The downstream neighbourhood, in contrast to the upstream one, is to some extent variable and next to the aforementioned genes others occur on species-specific basis (Figure 1A).

The analysis of interspecies variability of non-coding sequences adjacent to genes of *saoABC* operon demonstrated the existence of a few highly conserved motifs (Figure 1A). Upstream of *saoA* those are: −10 box TATAAT of the promoter recognised by sigma A (σ^A^) subunit of bacterial RNA polymerase (RNAP). The −35 box is present, though more variable among species. Next to the constitutive σ^A^ promoter another one is present. The promoter for the alternative sigma B (σ^B^) factor consisted of −35 and −10 boxes, GTTT and GGGTA, respectively [29]. Upstream of *saoB* there is only a motif of the extended −10 box (TGnTATAAT), which does not require the presence of −35 box for RNAP−σ^A^ holoenzyme binding. Upstream of *saoC* two unusual motifs occur. First is a pair of highly conserved sequences CTTGAAAT and TTAAACT, which resemble −10 and −35 boxes of a bacterial promoter but are not linked to any known staphylococcal alternative sigma factor, thus denoted here as a promoter for a putative sigma X (σ^X^) factor. The CTTGAAAT motif is highly conserved, as in 46 of 47 analysed species agrees with the given consensus sequence. On the other hand, the TTAAACT motif is more variable; however, the AAATC part is preserved in the case of 27 species, also including *S. aureus*. Markedly, the distance between the former and the AAACT part of the latter is 25 bp in the case of 37 species, again including *S. aureus*, the most optimal distance between −35 and −10 bacterial promoter boxes. Further downstream of the putative promoter sequence there is a span of c. 50 bp of moderately to highly conserved sequence. Within that span a palindromic motif AGACTGACGTTT-n14-AAACGTCAGTCT is present. In the case of *S. aureus* there is only one mismatch in the second repeat in respect to the given consensus.

Regarding the functional sequence analysis, an InterPro search detected any domain only in the case of SaoA, and it is the general stress protein 17M-like domain (GSP17M-like_dom, IPR025889) which is associated with stress proteins from *Bacilli*. Furthermore, none of the SaoA, SaoB and SaoC sequences hold any significant similarity to any known protein sequence of known function. When it comes to their variability among staphylococcal species, an unusual picture emerges (Figure 1B,C). The most conserved is SaoB, the length of which falls into the interval of 219–227 aa for analysed staphylococcal species. Pairwise sequence comparison reveals high sequence identity with the minimal value of 40% (mean A = 57%, harmonic mean H = 55%) and similarity with the min value of 60% (A = 73%, H = 72%). Less, however still quite conserved, is the sequence of SaoA (126–144 aa) with the min. identity of 21% (A = 46%, H = 40%) and min. similarity of 41% (A = 66%, H = 62%). Notably, the SaoC protein sequence manifests an entirely different and unique pattern of conservation and variability that has been observed before for a smaller number of staphylococcal species [26]. The variability is observed to a lesser extent for pairwise comparisons with minimal sequence identity of 32% (A = 47%, H = 45%) and similarity of 42% (A = 58%, H = 56%). What is highly variable about the SaoC sequence is predominantly its length (199–352 aa). Furthermore, SaoC variability is limited only to one region. Approximately 150 aa N-terminal fragment, and a short 12 aa C-terminal motif are highly conserved, whereas the remaining sequence between those two fragments varies substantially in respect to both sequence and length.

Unsurprisingly, the picture is entirely different when within-species variability is analysed. In this study we analyse in detail the variability only among *S. aureus* genomes, the species that is the main object of our research. However, our extended analysis showed that it holds true for other staphylococcal species as well (data not shown). For more than ten thousand *saoABC* occurrences found in different *S. aureus* genomes, SaoA, SaoB and SaoC protein sequences, with the exception of a total of 15 outliers, display nearly the same lengths, which are 135, 220 and 311–321 aa, respectively. Moreover, all possible pairwise comparisons showed that the protein sequences, with an exception of a total of 14 outliers, are characterised by minimal identity of 92% (SaoA and SaoB) and 82% (SaoC), as well as minimal similarity of 96% and 86%, respectively. Notably, in the case of *S. aureus*, the within-species variability of the SaoC protein sequence, and that of the *saoC* gene, remains relatively high and correlates with general phylogeny of this species, which has already proven to be as useful in genotyping as much as the widely used sequence of *rpoB* gene coding for the β subunit of bacterial RNAP [27].

**Figure 2:**
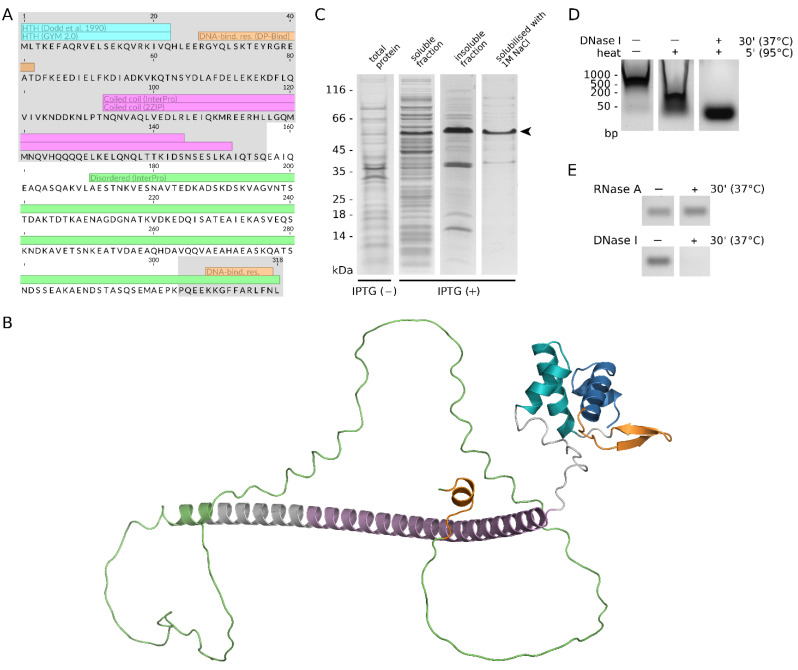
SaoC is a DNA-binding protein. (**A**) In the SaoC sequence from *S. aureus* it is possible to detect different DNA-binding motifs (HTH, skyblue; coiled coil, magenta) and extents of residues (orange) by using different available tools (GYM 2.0, NPS@ tool based on Dodd and Egan, DP-Bind, 2ZIP, InterPro Scan). Notably, all these motifs are located in the parts of SaoC that are highly conserved among different staphylococcal species, whereas the highly variable SaoC part is predicted to be of unordered structure (green). (**B**) Some of the predictions made by the aforementioned tools align with SaoC structure as modelled by AlphaFold. The N-terminal part starts with a HTH motif (blue), followed by a β-sheet corresponding to predicted DNA-binding residues (orange), and another, not predicted before, HTH motif (cyan). The region initially predicted as a coiled coil (magenta) is α-helical in the model, which may indicate the possible presence of a coiled coil tertiary structure. Most of the remaining part of SaoC remains unordered, similarly to the initial prediction (green). The C-terminal motif, which was predicted to contain DNA-binding residues (orange), is an exception as it is modelled as a short α-helix. (**C**) SaoC when produced in *E. coli* exhibits properties typical for DNA-binding proteins. It migrates slower in SDS-PAGE than expected by its molecular mass and forms aggregates that may be solubilised with 1 M NaCl. (**D**) The solubilised samples of SaoC contain nucleic acids as evidenced in agarose gel electrophoresis. They might be released from complexes with SaoC by thermal denaturation. If treated with DNAse I beforehand, short fragments are protected from digestion. (**E**) The short nucleic acid fragments are degraded by DNAse I but not by RNAse A, which shows they are in fact DNA fragments.

**SaoC is a DNA-binding protein**. Knowing that *saoABC* operon codes for three hypothetical proteins, we searched for known sequence motifs in their sequences, which pointed out the possibility that SaoC is a DNA-binding protein. Within the N-terminal part of SaoC, which is conserved among different staphylococcal species, it is possible to detect three DNA-binding motifs: an HTH (helix-turn-helix) motif, a coiled coil region and an extra span of residues putatively engaged in DNA binding. Interestingly, a short span of such residues is also detectable within the other conserved part of the SaoC sequence, the short C-terminus (Figure 2A). Notably, the variable part of SaoC is putatively of unordered structure. GYM 2.0 detects another HTH motif in different sites of the variable part in *S. aureus*, as well as in *S. arlettae*, *S. chromogenes*, *S. cohnii*, *S. condimenti*, *S. haemolyticus*, *S. hyicus*, *S. microti*, *S. rostri*, *S. schleiferi* and *S. schweitzeri*. However, this is not confirmed by NPS@ tool based on the method established by Dodd and Egan, and thus interpreted as false positive results. Some of the predictions made by the aforementioned tools align with SaoC structure as modelled by AlphaFold. In that model, SaoC N-terminal contains two HTH motifs, one aligning with the one initially predicted. These two are separated by a short β sheet spanning the region containing predicted DNA-binding residues. These three motifs together may form a protein-DNA interaction interface. Similarly to the initially made predictions, a substantial part of SaoC structure remains unordered (Figure 2B). All aforementioned motifs as well as disordered protein regions are demonstrated to take part in protein-DNA interactions [30], which suggests that SaoC might be a yet uncharacterised transcriptional factor. Indeed, SaoC capacity of binding to DNA is observed when it is over-produced in *E. coli* as a tag-less native protein (Figure 2C). The protein migrates in SDS-PAGE gel with a considerable shift in respect to its theoretical molecular mass (migration range 45–66 kDa, molecular mass 36 kDa). The identity of the protein was confirmed by Western blotting and Edman degradation of its N-terminus. Upon sonication of bacterial suspension, and subsequent centrifugation, most of the over-produced protein remains in the insoluble fraction; however, it remains not as inclusion bodies but DNA-protein aggregates soluble in buffers containing 1M NaCl. These are features displayed by some DNA-binding proteins [31,32,33]. Samples of such solubilised protein contain nucleic acids as detected in ethidium-bromide-stained 1% TAE agarose gel. These nucleic acids migrate faster upon heat denaturation of a sample, which suggests their release from complexes with protein molecules. When treated with DNAse I before heat denaturation, short nucleic acid fragments still remain in the sample, most likely due to protection by the bound protein (Figure 2D). Such free fragments are degraded by DNAse I but not RNAse A, which demonstrates they are DNA fragments (Figure 2E). The same treatment of *E. coli* cultures either containing an empty expression vector or overproducing a DNA-non-binding GST (glutathione S-transferase) protein yields samples with no detectable nucleic acids. This confirms that SaoC is in fact a DNA-binding protein.

**Figure 3:**
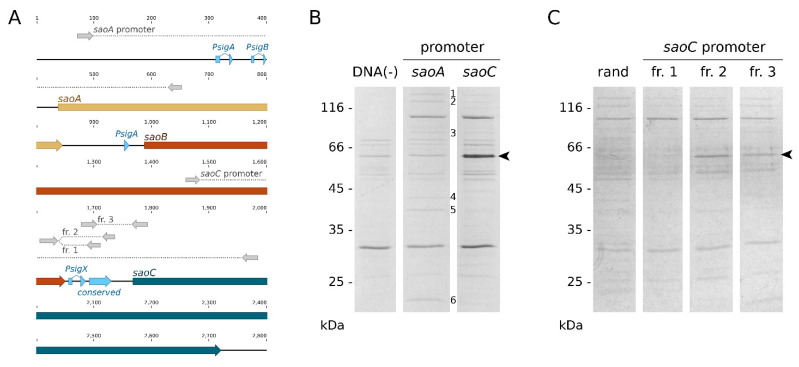
SaoC binds within the uncharacterised conserved motif upstream of its own gene. (**A**) The scheme showing which fragments of *saoABC* operon were used in the experiment and which conserved motifs they encompassed. Next to two major regions covering vast extents of putative promoter regions of *saoA* and *saoC*, three shorter fragments of the latter were used to narrow down the region bound by SaoC. (**B**) SDS-PAGE of protein samples from DNA pull-down assays performed on the two major putative promoter regions of *saoA* and *saoC* and whole-cell *S. aureus* lysates. When compared to the control without DNA, the major fragment of *saoC* promoter is bound by SaoC protein as confirmed by mass spectrometry. In the case of the *saoA* promoter region, no evident binding is detectable in applied conditions. The identity of a few differentiating bands (1–5) points at general DNA-binding proteins or their fragments (1, single-stranded DNA-binding protein; 2, 3′–5′ exoribonuclease YhaM; 3, type I DNA topoisomerase; 4, DNA polymerase I; 5, RNAP β’ subunit; 6, RNAP β subunit). (**C**) SDS-PAGE of protein samples from DNA pull-down assays performed correspondingly on the three shorter fragments of the putative *saoC* promoter region. Only the fragments containing the uncharacterised conserved region are bound by SaoC. The control used here was a randomly shuffled fragment of the *saoC* putative promoter sequence.

**SaoC binds to a sequence motif upstream of its own gene**. In order to determine factors that may bind the conserved, uncharacterised sequences located upstream of *saoC* (Figure 1A and Figure 3A), we chose to carry out pull-down assays with different fragments of *saoABC* operon non-coding sequences (Figure 3A) immobilised on magnetic beads and whole cell lysates obtained from *S. aureus* RN4220. Whilst only general DNA-binding proteins can be detected for putative *saoA* promoter region (Figure 3B) when compared to no-DNA control in the conditions applied, in the case of the putative *saoC* promoter region a strong band appears. As evidenced by mass spectrometry, that band corresponds to the SaoC protein itself, which means the protein binds to regulatory sequences that are present upstream of its own gene. When shorter fragments of the putative *saoC* promoter region are used that encompass different conserved motifs upstream of the gene (Figure 3A), and are compared to a shuffled DNA fragment, the binding site might be narrowed down to the region containing the uncharacterised conserved motif, which includes the palindromic sequence AGACTGACGTTT-n_14_-AAACGTCAGTCT (Figure 3C).

**Figure 4:**
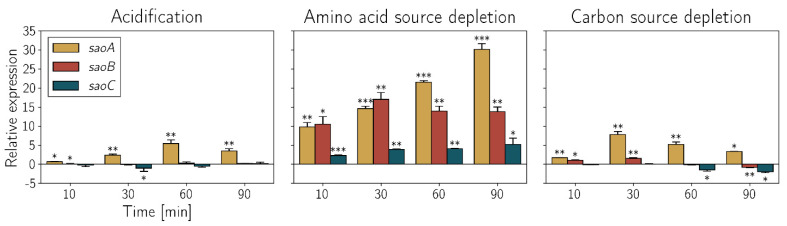
Expression changes of *saoABC* operon genes in *S. aureus* CH91 strain manifested reproducibly in three distinctive stress conditions, including acidification as well as depletion of amino acid or preferential carbon sources, measured with RT-qPCR (*n* = 3) in reference to transcripts of gyrase B (*gyrB*). In all conditions, particularly elevated expression of *saoA* is observed. Similar changes are observed for *saoB* in the case of amino acid source depletion. Preferential carbon source depletion leads to decreased expression of *saoB* and *saoC.* * *p* ≤ 0.050, ** *p* ≤ 0.010, *** *p* ≤ 0.001.

**Expression of *saoABC* genes is elevated in stress conditions**. Revealing the presence of σ^B^ promoter upstream of the whole *saoABC* operon suggested its participation in the stress response. In order to confirm that suspicion we tested the expression of all three operon genes in *S. aureus* in different stress conditions: acidification, antibiotic exposure, depletion of the amino acid source or the preferential source of carbon, heat stress, osmotic stress, oxidative stress and salinisation. Among all these conditions we observed changes of the expression pattern of *saoABC* genes only in the case of acidification, depletion of the amino acid source or the preferential source of carbon (Figure 4). The expression of *saoA* is particularly elevated by acidification and depletion of the preferential carbon source, whilst depletion of the amino acid source alters, in a similar manner, the expression of *saoB* as well. In the case of depletion of the preferential source of carbon, a decrease in the expression of *saoB* and *saoC* is observed.

**Figure 5:**
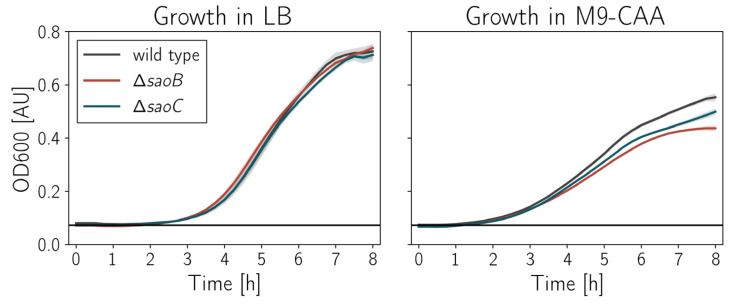
Growth dynamics of RN4220 strain knockout mutants of *saoABC* operon genes. In rich media there is not any difference in growth dynamics (plot on the left). However, in less optimal M9-CAA, Δ*saoB* and Δ*saoC* manifest slower growth and reach lower densities by around 20 and 10%, respectively, when compared to wild type RN4220 (plot on the right). The standard deviation of measurements (*n* = 3) is depicted as shaded bands.

**Mutations in *saoABC* operon alter growth dynamics**. In order to delve deeper into the link between *saoABC* operon and nutrient starvation, we obtained knockout mutants for *S. aureus* RN4220 for each of the operon genes by insertion of group II introns. These mutant strains are referred further to as Δ*saoA*, Δ*saoB* and Δ*saoC*. When grown in rich media such as TSB or LB, they manifest the same growth dynamics. However, in the case of M9-CAA, a more basic medium that is not optimal for staphylococci, Δ*saoB* and Δ*saoC* grow slower and reach lower densities in the stationary phase when compared to the wild type RN4220 strain (Figure 5).

**Figure 6:**
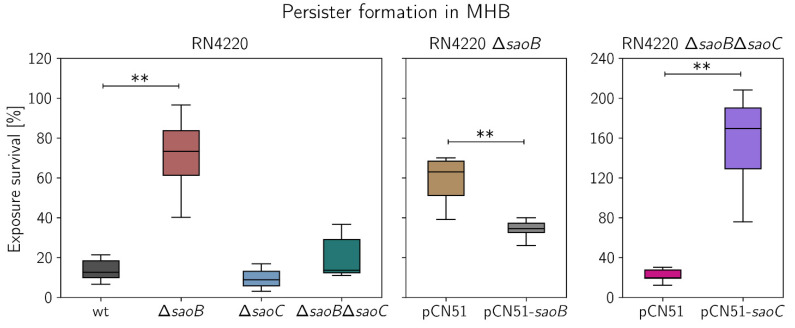
Persister formation by RN4220 strain knockout mutants of *saoABC* operon genes. Significant differences (*n* = 6) are observed only between wild type (wt) RN4220 and RN4220 Δ*saoB* (plot on the left). The null mutant is characterised by around 4-fold increased survival during exposure to penicillin. The effect is to some extent reversed by complementation of RN4220 Δ*saoB* with pCN51 plasmid vector containing *saoB* controlled by a cadmium-inducible promoter (plot in the middle). Importantly, no statistically significant increase in survival is observed between the wild type RN4220 and RN4220 Δ*saoC*, or the double mutant RN4220 Δ*saoB*Δ*saoC* (plot on the left). However, cadmium-induced expression of *saoC* in the double mutant significantly increases persister formation (plot on the right). ** *p* ≤ 0.010.

**Disruption of *saoB* and induction of *saoC* expression boost persister formation**. Given the fact that expression of *saoABC* operon genes is altered by nutrient deficit, the mutants Δ*saoB* and Δ*saoC* display suppressed growth dynamics, and since persister formation phenomenon has been persuasively linked to starvation by multiple research groups [10], we tested Δ*saoA*, Δ*saoB* and Δ*saoC* strains, as well as the double mutant Δ*saoB*Δ*saoC*, for their potential to produce persisters during exposure to penicillin (Figure 6). Once again, we did not observe any difference between the wild type RN4420 strain and Δ*saoA*, and unexpectedly between the wild type and Δ*saoC*. In contrast, the Δ*saoB* strain displays around 4-fold higher persister fraction during exposure to the antibiotic when compared to wild type RN4220. Markedly, the difference does not appear in the case of Δ*saoB*Δ*saoC* double mutant. Complementation of Δ*saoB* with pCN51 plasmid vector containing *saoB* controlled by a cadmium-inducible promoter leads expectedly to a decrease in survival when compared to Δ*saoB* with empty pCN51. Markedly, the survival of Δ*saoB*Δ*saoC* double mutant, when production of SaoC is induced from pCN51 containing *saoC*, is increased significantly and the most among all analysed cases.

**Figure 7:**
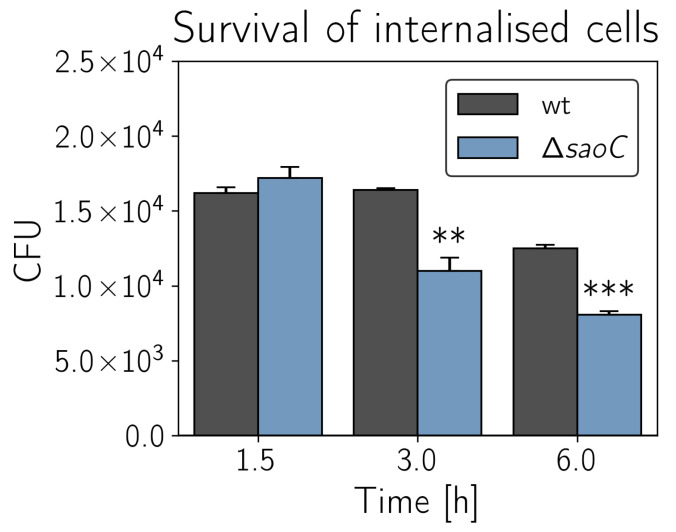
Survival of RN4220 strain knockout mutant Δ*saoC* in human dermal fibroblasts. RN4220 Δ*saoC* reproducibly manifests decreased survival of cells internalised to human fibroblasts (*n* = 3). The survival is lower by 25–30% to that of the wild type RN4420 in the third and sixth hour after exposure of fibroblasts to bacterial cells. ** *p* ≤ 0.010, *** *p* ≤ 0.001.

**Disruption of *saoC* decreases intracellular survival in fibroblasts**. In the context of the observed differences in persister formation, we decided to assess to what extent genes of *saoABC* operon may influence the survival of staphylococci internalised to human cells. Fibroblasts were chosen to model survival of staphylococcal cells for two reasons. Firstly, fibroblasts are abundant in the human body and often exposed to invading bacteria, especially in the case of damaged skin or mucous membranes. Secondly, as non-professional phagocytes they are not specialised in killing internalised bacterial cells, which allows for assessment of the efficiency at which bacteria evade the immune response by internalisation. Similarly to previously described results, we did not observe any difference between the wild type RN4420 strain and Δ*saoA*. In the case of Δ*saoB*, any changes if present were minor and irreproducible. Only Δ*saoC* manifests decreased survival when internalised to human fibroblasts (Figure 7), which is 25–30% less than that for the wild type RN4420 strain in the third and sixth hour after exposure of fibroblasts to bacterial cells.

**Figure 8:**
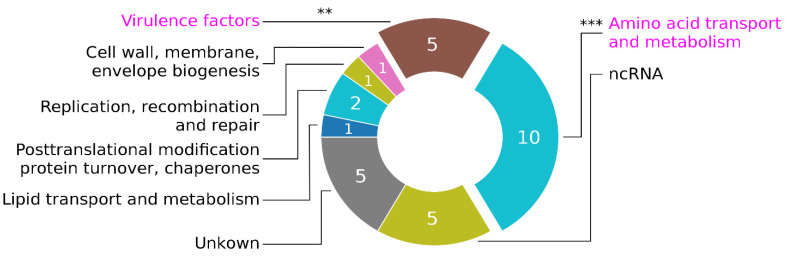
Representation of genes involved in different cellular processes among those differentially expressed in the logarithmic growth phase in RN4220 Δ*saoC* when compared to the wild type. Although a small number of differences (30 loci) may be observed, a general trend emerges that differentially expressed loci are mostly linked to basic metabolism or virulence. Genes related to amino acid transport and metabolism, as well as virulence factors, are significantly over-represented. The number of differentiating loci is given on each slice. ** *p* ≤ 0.010, *** *p* ≤ 0.001.

**SaoC protein influences expression of genes related to basic metabolism as well as virulence**. As an attempt to link phenotypic changes in *saoABC* operon mutants, which we described in preceding paragraphs, to functions of the DNA-binding protein SaoC, we decided to undertake an RNA-Seq experiment. When the whole transcriptome of RN4220 Δ*saoC* is compared to the one of the wild type, a small number of differences (30 loci, 25 when operons are counted as one) is observed in the logarithmic growth phase (Table 1), and even smaller in the late phase (6 loci, Table 2). However, next to the important fact that in both growth phases transcription of *saoC* locus is increased in the absence of functional SaoC (see Supplementary Figure S1 for details), a general trend emerges that differentially expressed loci/operons are linked to basic metabolism or virulence. It is particularly pronounced in the log-phase, in which case there is a statistically significant over-representation of genes involved in amino acid transport and metabolism as well as of those encoding different virulence factors (Figure 8). Within the former group, all genes with decreased expression (up-regulated by SaoC) are either amino acid transporters or genes involved in arginine uptake and biosynthesis: argininosuccinate synthase and argininosuccinate lyase (*argG*, *argH*), arginine-ornithine antiporter and ornithine carbamoyltransferase (*arcD*, *arcB*). Whereas genes with increased expression (down-regulated by SaoC) are engaged in methionine and glutamate synthesis: homoserine O-acetyltransferase (*metX*) and the large subunit of glutamate synthase (*gltB*). Regarding virulence factors, in RN4220 Δ*saoC* decreased expression (directly or indirectly up-regulated by SaoC) is observed in the case of bicomponent leukotoxin LukH/LukG (*lukH*, *lukG*) and superantigen-like protein Ssl11 (*ssl11*), whereas increased expression (directly or indirectly down-regulated by SaoC) is observed for triacylglycerol lipase Sal1 (*gehA*) and VraX protein (*vraX*).

## 3. Discussion

The *saoABC* operon occurs in most of the known staphylococcal species, and importantly in all that are relevant for either clinical or veterinary reasons, including *Staphylococcus aureus*. The within-species penetrance of the operon reaches 100%, which means if the operon is found in a given species that all strains of that particular species are carriers. Moreover, the variability of the operon sequence correlates with phylogenetic relations among *S. aureus* strains [27], which means that its evolutionary history is long. Such high prevalence and evolutionary conservation suggest that the functions of *saoABC* operon are highly likely of substance. Nonetheless, the role of *saoABC* operon has remained unknown to date. To address this problem we undertook an investigation, the results of which show that functions provided by *saoABC* operon are linked to the response to nutritional stress and, consequently, to the virulence of staphylococci.

The earliest reports linking *saoABC* operon with the response to stress are those aiming at regulon determination for the staphylococcal alternative σ^B^ factor of bacterial RNAP [35,36]. That being said, our study stems from the one on *pemIK-Sa1* toxin–antitoxin system, during which we found that *saoC* is significantly under-represented in the sequence motif recognised by PemK-Sa1 RNase [27,28]. Similarly to the latter, reports on σ^B^ did not provide any inquiry into *saoABC* operon itself. It is simply possible to see in their results that *saoA* expression is elevated in presence of σ^B^. In Northern blotting results provided by Gertz et al., one may also notice that the probe for *saoA* gives three signals with transcript lengths corresponding to *saoA* transcribed alone, together with *saoB* or a transcript of the whole operon. These notions correspond to the basic facts on *saoABC* operon presented in this study (Figure 1A). One of its promoters, located upstream of *saoA*, is a promoter of σ^B^. The variety of regulatory sequences present in *saoABC* operon may facilitate alternative transcript generation and would explain why the expression of its genes may be changed independently from each other (Figure 4, Table 1 and Table 2). Apart from the existence of transcripts suggested by the results by Gertz et al. [35], it is expected to observe *saoBC* transcript synthesised from the second σ^A^ promoter or *saoB* transcript alone when the transcription of *saoC* is repressed by SaoC protein binding to the conserved palindromic sequence (Figure 3, Table 1 and Table 2). The question remains as to whether *saoC* can be transcribed alone. That would be potentially an option if, as uncovered in this study, the highly conservative promoter-like sequence motifs upstream of *saoC* were, as we suggest, a promoter of a yet unknown staphylococcal alternative σ^X^ factor of bacterial RNAP (Figure 1A). This hypothesis gains more appeal when two more facts are taken into account, namely that the putative, highly conserved −35 box CTTGAAAT corresponds ideally to the consensus sequence for −35 box for *E. coli* heat-shock σ^32^ factor, which is CTTGAAA [37,38], and the transcription of *saoC* locus can be elevated independently of *saoA* and *saoB* (Supplementary Figure S1). Nevertheless, currently in the matter of *saoABC* operon transcription there are only results published by Gertz et al., and thus we only known for certain there are alternative transcripts that encompass *saoA*. Generation of other transcripts of *saoABC* operon proposed here, as well as the function of the conserved hypothetical σ^X^ promoter sequence, still await detailed investigation. However, based on existing data, including ours, it is clear that the transcriptional regulation of *saoABC*, an operon of three relatively short genes, is undoubtedly complex.

Another non-typical characteristics of *saoABC* operon is the conservation pattern and length variability of SaoC protein (Figure 1B,C). High conservation among different staphylococcal species of the N-terminal part and the short C-terminal fragment of SaoC drew our attention previously, as it pointed to *saoC* as a good target for genotyping [26,27]. In the context of this study, it is worth noting that both conserved parts of SaoC are where DNA-binding motifs are likely located (Figure 2A). Hence, the sequence motif bound by SaoC is likely similar among different staphylococcal species despite the high variability of a part of SaoC sequence. That would also explain why SaoC binding site was found within the highly conserved among staphylococcal species palindromic sequence motif (Figure 3). The reason behind high variation of the linker between the conserved parts of SaoC, in terms of both length and the sequence variability, remains to be elucidated.

The presence of a σ^B^ promoter upstream of *saoABC* operon, which makes it a part of σ^B^ regulon [35,36], indicates existence of a link between functions of *saoABC* operon and the response to stress. Involvement of σ^B^ factor in the general stress response in Gram-positive bacteria is well documented [39,40]. Importantly, σ^B^ is one of three known RNAP alternative sigma factors in *S. aureus*. The other two are σ^H^ and σ^S^. The former is related to natural competence, the latter is implicated in survival in prolonged stress conditions [41,42]. Nevertheless, σ^B^ is the major player in the staphylococcal regulatory mechanism of the stress response, and consequently *saoABC* operon seems to be another element of this mechanism. Indeed, we showed specific changes in the expression of genes of *saoABC* operon when bacterial cells were exposed to acidification as well as depletion of preferential carbon or amino acid source (Figure 4). The response to stress stimuli quite often decreases bacterial metabolism, which facilitates survival of bacterial cells in adverse conditions. It may lead to biofilm formation as well as persister phenotype [7,10]. In the course of our study, we were able to link *saoABC* operon to bacterial survival in adverse conditions and demonstrate how it influences persister formation. We observed decreased growth dynamics of RN4220 Δ*saoB* and Δ*saoC* mutants in non-optimal media, specific changes in persister formation in the case of RN4220 Δ*saoB*, Δ*saoC* and double Δ*saoB*Δ*saoC* mutants (also when supplemented with cadmium-induced *saoB* and *saoC*), as well as decreased intracellular survival of RN4220 Δ*saoC* mutant in fibroblasts (Figure 5, Figure 6 and Figure 7). Combining with the preliminary study on SaoC regulon in RN4220 (Figure 8, Table 1 and Table 2) a picture emerges suggesting that *saoABC* operon is mainly linked to the response to amino acid starvation, and in such conditions facilitates bacterial cell survival and promotes persister formation.

Regarding the influence of SaoC on the expression of genes engaged in amino acid transport and metabolism (Table 1), it appears that in the absence of SaoC the expression of genes engaged in amino acid uptake and arginine biosynthesis via the urea cycle is decreased. It seems a logical course of action, during a limitation of the amino acid/nitrogen source, to increase the number of transporters, and consequently the uptake of amino acids, and simultaneously down-regulate amino acid-dependent metabolic pathways, such as the synthesis of methionine and glutamate. Elevated arginine biosynthesis during starvation might at first appear unexpected, but not when the fact that arginine is used as an alternative carbon source when the preferred one is exhausted is taken into consideration. That links SaoC yet again with the metabolic response to preferred carbon source limitation, and thus with functions of CcpA in arginine metabolism via the urea cycle [18,43].

The influence of SaoC on the expression of virulence factors (Table 1) should be considered in the aforementioned context since the metabolic state of a bacterial cell strictly determines whether it switches to invasive or latent phenotype. A number of staphylococcal transcription factors are a good example of such a phenomenon. These include: CcpA, PurR and CodY, primarily responsible for metabolic control of carbon catabolism, purine biosynthesis and nitrogen metabolism, respectively, though also linking the metabolic state a bacterial cell with virulence [23]. RNA-Seq results suggest that SaoC presence may contribute to the increase in production of bicomponent leukotoxin LukH/LukG and superantigen-like protein Ssl11, both responsible for immune evasion. The former tandem is a pore-forming toxin leading to lysis of neutrophils [44], the latter affects their motility [45]. That agrees with the general pathogenesis progress during whose late stages bacterial cells experience nutrient stress, and in consequence start production of toxins and switch to the invasive phenotype. On the other hand, SaoC likely represses the expression of SAL-1 lipase and VraX protein. The former implicated in immune evasion and promoting biofilm formation [46]. The latter is also linked to immune evasion, the cell wall stress response and general increase of virulence as well [34]. That again may suggest that SaoC plays the main role in late colony growth stages, in which nutrient deficit promotes biofilm dispersal and the spread of bacterial cells to secondary infection sites. Nonetheless, the interplay of different virulence factors in different environmental contexts, as well as still limited knowledge on the exact mechanisms of SaoC–DNA interaction, make it rather difficult to come up with a compelling hypothesis on how SaoC influences the expression of aforementioned virulence-related genes and shapes the phenotype and behaviour of staphylococcal cells. The results we present indicate that SaoC functions as a transcription factor linking the metabolic state of a bacterial cell with manifestation of its virulence. The general conclusion is that SaoC likely plays an important role primarily in the response to amino acid/nitrogen starvation, whilst affecting in consequence, staphylococcal virulence and persistence. A deeper understanding of mechanisms underlying the observed dependencies will require further studies. Notably, among genes with decreased expression in the log-phase in the absence of SaoC there is also one non-coding RNA (srn_9200_sRNA101). Its expression fold-change of almost −50 is remarkable, however nothing else can be said since the function of this ncRNA is currently unknown.

The exact mechanism according to which SaoC influences gene expression remains also unknown. Although we found that it binds to the palindromic motif upstream of its own gene, we were unable to find a similar motif in any promoter region in the *S. aureus* genome. That might seem puzzling; however, similar observations have been made for the recently described MbtS [12]. Markedly, the similarities between SaoC and MbtS extend further. Their genes are located as the last ones in three-gene operons. However, MbtS is on average smaller (189 aa) than SaoC (199–352 aa), and sequence homology between them is poor (Supplementary Figure S2). In addition, our preliminary data also suggest that, similarly to MbtS, SaoC is a membrane-bound transcription factor (Supplementary Figure S3). In the case of MbtS and two associated hypothetical proteins the presence of transmembrane helices is predicted, which is not the case for SaoA, SaoB or SaoC. Moreover, in contrast to SaoC, it has not yet been determined what the possible role of MbtS is. The aforementioned results and analogies again suggest that SaoC functions as a transcription factor. However, the exact mechanisms according to which both SaoC and MbtS influence gene expression remains to be determined, it is highly likely that the continuation of research focused on one will contribute to a better understanding of the other.

**Figure 9:**
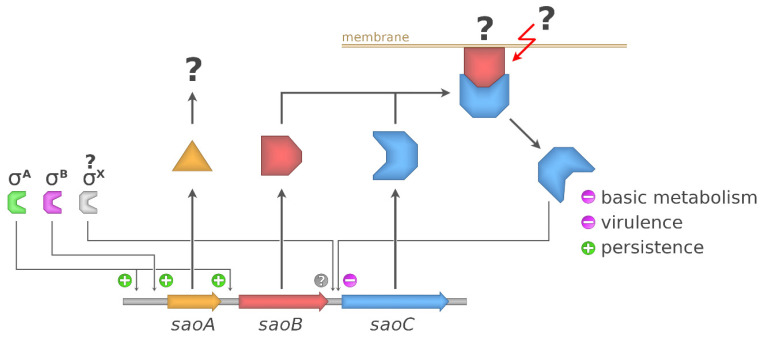
Possible regulatory pathways governing functions of *saoABC* operon. Promoter sequences of *saoABC* operon, next to constitutive promoters for σ^A^, contain also one for σ^B^. The latter likely increases transcription in stress conditions, not to turn on the SaoC-related system but in order to regenerate SaoA and SaoB proteins and deactivate released SaoC. Functional links between *saoABC* operon and other, yet uncharacterised, regulatory systems may exist, which is indicated by the presence of conserved but unknown sequence motifs upstream of *saoC*. It is highly likely that SaoC is a transcription repressor that autoregulates the transcription of its own gene in a negative feedback loop. SaoC is engaged in regulation of the expression of genes related to basic and mostly protein metabolism, but also of genes of virulence factors. The general result of its action seems to be metabolic suppression, virulence attenuation and persister formation. SaoB is highly likely an antagonist of SaoC, and might be a membrane-attached protein that keeps SaoC inactive in a membrane-bound state. Functions of SaoA still remain unknown. The role of SaoA and the nature of SaoB, as well as molecular mechanisms underlying interactions among them and SaoC, are yet to be determined.

Taking all facts together, it is still not possible to propose any binding view on the mechanisms underlying the functions of *saoABC* operon and its products: SaoA, SaoB and SaoC proteins. Nevertheless, from the data gathered by us a coherent picture is emerging (Figure 9). It is unsurprising that the functions of *saoABC* operon are entwined with those of another global stress response system, well-known for Gram-positive bacteria, namely the alternative σ^B^ factor of bacterial RNAP (Figure 1A). Moreover, there are possible functional links between *saoABC* operon and other, yet unrevealed, regulatory systems. Such a hypothesis seems to be supported by the presence of conserved sequence motifs of unknown functions in the putative promoter region upstream of *saoC* (Figure 1A). SaoC plays an important role in the stress response to nutrient deficit. Highly likely it is a transcription repressor that autoregulates the transcription of its own gene in a negative feedback loop (Table 1 and Table 2, Figure 1A and Figure 3). Nutrient deficit is a global problem for a bacterial cell and thus, SaoC regulates the expression of genes not only directly engaged in the basic and mostly protein metabolism, but also of genes of virulence factors (Table 1 and Table 2, Figure 8). These finally contribute to metabolic suppression, persister formation and likely virulence attenuation (Table 1, Figure 5, Figure 6, Figure 7 and Figure 8). Whilst the functions of SaoA still remain a mystery, SaoB is highly likely an antagonist of SaoC, as either the absence of SaoB or overexpression of SaoC in the absence of SaoB result in elevation of persister formation (Figure 6). Such a role of SaoB remains consistent with the presence of σ^B^ promoter and the negative feedback loop in transcription of *saoC* (Supplementary Figure S1). The loop would inhibit *saoC* transcription upon SaoC release in stress conditions by yet unknown factors, whilst transcription of *saoA* and *saoB* would be elevated by stress-activated σ^B^ (Table 1 and Table 2, Figure 4). That would, in consequence, lead to σ^B^-dependent elevated production of SaoA and SaoB, and subsequent sequestration of initially released SaoC by the regenerated amount of SaoB. Hence, we speculate that σ^B^ is not indispensable for triggering the SaoA/B/C regulatory system but on the contrary, it only speeds up system deactivation by providing accessory, stress-induced promoter for *saoA* and *saoB* transcription. Regarding SaoA, it possibly plays some accessory role, either related to gene expression of *saoABC* operon or the interaction between SaoB and SaoC. SaoB might be a membrane-attached protein, either directly or indirectly by an unknown component, and that, apart from keeping SaoC inactive, would keep it in a membrane-bound state as well. Nevertheless, the role of SaoA and the nature of SaoB proposed here are speculative, and together with the exact mechanisms propelling the interactions of these two proteins with SaoC are yet to be determined. We believe that further studies on *saoABC* operon will bring equally interesting results to those obtained by us so far, and contribute to better understanding of the stress response of staphylococci as well as links joining it to their virulence.

## 4. Materials and Methods

**Plasmids and primers used in the study**. For protein expression purposes, the following plasmids were used: pETDuet-1 (Novagen, Darmstadt, Germany), pGEX-5T [47], pCN35, pCN51 and pCN68 [48]. Plasmid constructs, unless otherwise described, were obtained either by the common restriction cloning protocol or ligase independent cloning. All the plasmid constructs and primers used in the study are described in detail in Supplementary Table S1. Restriction cloning PCR products were obtained using RUN DNA Polymerase (A&A Biotechnology, Gdynia, Poland), purified with Clean-Up Concentrator (A&A Biotechnology, Gdynia, Poland) and digested with respective enzymes (Thermo Scientific, Waltham, MA, USA). Plasmids were isolated and purified from *Escherichia coli* TOP10 (Invitrogen, Waltham, MA, USA) with Plasmid Mini (A&A Biotechnology, Gdynia, Poland). Upon digestion and electrophoresis, DNA fragments were incised and isolated from agarose gel using Gel-Out Concentrator (A&A Biotechnology, Gdynia, Poland)and ligated using T4 DNA Ligase (Thermo Scientific, Waltham, MA, USA). All procedures were carried out in accordance with the recommendations of the manufacturers. For ligase independent cloning, Phanta Max Super-Fidelity DNA Polymerase (Vazyme, Nanjing, China) was used both to obtain a gel-purified PCR product of the insert, and to carry out the second PCR reaction with the product (megaprimer) and the target plasmid as described by Van Den Ent and Löwe [49].

**Data analysis and visualisation**. Graphical representations of sequence motifs and sequence conservation were prepared in CLC Main Workbench (ver. 21.0.3, CLC Bio, Aarchus, Denmark). Data processing and statistical analysis, if not otherwise indicated, as well as data plots were prepared in Jupyter Notebook application (ver. 4.7.1) using in-house Python (ver. 3.8.5) scripts utilising Matplotlib (ver. 3.2.2), Pandas (ver. 1.1.4), Numpy (ver. 1.19.4) and SciPy (ver. 1.5.2) libraries. The SaoC structure model was rendered with PyMOL (ver. 2.5.0). Final figures were prepared in GIMP image editor.

**The analysis of *saoABC* operon prevalence and diversity**. Genomic sequences of bacteria from *Staphylococcus* genus, 13,440 in total, including complete and whole genome shotgun ones, were obtained from the GenBank database (accessed on 13 September 2019). Subsequent steps utilised BLAST tools 2.10.1+ [50]. All possible open reading frames were extracted from genomic sequences, translated and compiled into a BLAST database. The databases were searched using the PSI-BLAST tool (psiblast) and SaoA, SaoB and SaoC sequences obtained from *S. aureus* as query sequences. The most frequently occurring sequences for each species from the *Staphylococus* genus were extracted from the genomic ones, and *saoABC* operon genes were annotated manually based on the presence of ribosome-binding sites. These representative, annotated sequences of *saoABC* operon derived from different staphylococcal species are provided in a supplementary multiple sequence GenBank file (saoABC.gb). Translations of manually curated annotations were used as representative sequences to re-search the database of protein sequences obtained from the analysed genomes using the Protein BLAST tool (blastp). Only fragments of matches completely covering query sequences and derived from genomes that contained genes coding for all three proteins were used to assess SaoA, SaoB and SaoC sequence diversity across all staphylococcal species. Multiple-sequence alignments of representative *saoABC* operon, as well as SaoA, SaoB and SaoC protein sequences were constructed using Clustal Omega (ver. 1.2.4) [51]. The alignments are provided in Supplementary Files saoABC_aln.fna, SaoA_aln.faa, SaoB_aln.faa and SaoC_aln.faa.

**Search for sequence motifs and domains**. The protein sequences analysed in this study were scanned for the presence of a variety of sequence motifs and domains using the InterPro Scan tool (InterPro database accessed on 10 October 2021) and others, such as GYM 2.0 [52] and a tool provided by NPS@ based on the method by Dodd and Egan for HTH motif prediction [53,54], DP-Bind for prediction of DNA-binding residues [55], 2ZIP for leucine zippers prediction [56], and AlphaFold (database accessed on 1 June 2022) for obtaining SaoC structure prediction (UniProt accession number Q2G0Z2) [57].

**Preparation of s*aoABC* operon gene mutants**. The mutants of *saoABC* operon genes investigated in this study, referred to as Δ*saoA*, Δ*saoB* and Δ*saoC*, were obtained either by using pKOR1 plasmid for allelic replacement, as described in the original research by Bae and Schneewind [58], or the TargeTron™ Gene Knockout System and re-targeted pNL9164 (Sigma-Aldrich, Darmstadt, Germany). The latter facilitates the use of group II intron Ll.LtrB-ΔORF derived from *Lactococcus lactis* to obtain site-specific gene disruptions by insertion of 0.9 kb fragments devoid of any coding sequences. The procedures were undertaken in accordance with the protocol provided by the manufacturer and the original work of Yao et al. [59]. In the initial stages of the presented research, Δ*saoA* and Δ*saoC* mutants were obtained with pKOR1 and served for experiments on growth dynamics and intracellular survival in fibroblasts. To repeat these experiments and undertake the remaining ones, Ll.LtrB-ΔORF was used for its higher efficiency and ease of use for site-specific mutant generation. Using this method, Δ*saoB* and Δ*saoC* mutants were obtained and used/re-used for all of the experiments described in this work. It should be stressed that the initial experiments undertaken on Δ*saoC* mutants obtained with either pKOR1 or Ll.LtrB-ΔORF, that is the analysis of growth dynamics and intracellular survival in fibroblasts, gave the same results.

**Strains used in the study and growth conditions**. Unless stated otherwise all *S. aureus* CH91 (a generous gift from Prof. S. Takeuchi, Fukui Prefectural University) and RN4220 [60] were grown on solid TSA or in 20 mL of liquid TSB medium (Sigma-Aldrich, Darmstadt, Germany) at 37 °C, in the case of the liquid cultures, with 180 RPM shaking. *E. coli* strains were grown similarly; however, in LBA and LB media (Sigma-Aldrich, Darmstadt, Germany). *E. coli* TOP10 (Invitrogen, Waltham, MA, USA) was used for cloning and BL21(DE3) Gold (Novagen, Darmstadt, Germany) for protein expression. Fresh cultures were prepared by 1:100 dilution of overnight ones. The growth dynamics were monitored in 96-well plates in Sunrise plate reader (Tecan, Mannedorf, Switzerland) incubated at 37 °C with mixing before every measurement taken at 5 min intervals.

***S. aureus* cultures grown in stress conditions**. Bacteria were grown overnight in 20 mL of TSB medium at 37 °C with 180 RPM shaking. Fresh cultures were prepared by 100-fold dilution of the overnight one. The culture growth was continued in the same conditions until OD_600_ reached 0.5–0.6. Next, cultures were subjected to antibiotic exposure, osmotic and oxidative stress, and salinisation by being respectively supplemented with the following agents at the final concentrations: 1 or 10 μg/mL erythromycin, 1 M sorbitol, 10 mM H_2_O_2_, 1 M NaCl. In the case of heat-shock treatment, respective cultures were transferred to a water incubator, and growth was continued at 43 °C. Cultures subjected to acidification were centrifuged at 5000 RCF at room temperature for 10 min, and subsequently resuspended in 20 mL of fresh, pre-warmed portions of TSB medium with pH altered to 5.0. For investigation of the effects of amino acid and preferential carbon source depletion, bacteria were grown in the same manner in M9-CAA supplemented with glucose [61] that subsequently was changed to one devoid of casamino acids or glucose, respectively. Samples for RNA isolation were collected directly before introducing stress agents (t = 0) and at 10, 30, 60 and 90 min of growth continuation in stress conditions.

**Preparation of whole-cell lysate of *S. aureus***. In order to obtain whole-cell lysate, *S. aureus* RN4220 was grown overnight in TSB at 37 °C with 180 RPM shaking. A fresh 600 mL culture was prepared by diluting the overnight one with TSB medium at ratio 1:100 and incubated in the same conditions until it reached OD_600_ around 3.0. Bacteria were centrifuged at 5000 RCF at 4 °C for 30 min, washed twice by resuspension in 80 mL of PBS and 15 min centrifugation at 5000 RCF at 4 °C, and finally resuspended in 30 mL of lysing buffer (10 mM Tris-HCl; 200 mM NaCl; 1% *v/v* Triton X-100; 10 mM MgCl_2_; 10 mM β-mercaptoethanol; pH 8.0) supplemented with EDTA-free Complete Protease Inhibitor Cocktail (Roche Diagnostics, Basel, Switzerland) in line with the manufacturer’s recommendations. The bacterial suspension was divided in 1 mL portions and mechanically disrupted with 700 mg of unwashed glass beads (0.1 mm, Sigma Aldrich, Darmstadt, Germany) using the Precellys instrument (Bertin Instruments, Montigny-le-Bretonneux, France) according to the following programme: 4500 RPM speed for 10 cycles, 90 s each with 1 s break. The mechanical lysis was applied a total of four times, the samples were cooled on ice for 10 min after each repetition. Finally, the samples were centrifuged at 16,000 RCF at 4 °C for 5 min, the supernatant collected, supplemented with glycerol to the final concentration of 5%, and stored at −20 °C. The final protein concentration in such a preparation, measured with the BCA method, oscillated around 1.5 mg/mL.

**DNA-protein pull-down assay**. DNA fragments were obtained by using *S. aureus* genomic DNA or randomly shuffled fragments of the *saoC*-upstream sequence (Supplementary Sequence 1, GenScript, Leiden, The Netherlands) in a 30-cycle PCR with biotinylated forward primers (Genomed, Warsaw, Poland) and Phusion High-Fidelity DNA Polymerase (Thermo Scientific, Waltham, MA, USA) in accordance with the manufacturer’s recommendations. PCR products were subjected to electrophoresis in 1% agarose gel, and isolated from gel using Gel-Out Concentrator (A&A Biotechnology, Gdynia, Poland). For each binding assay, a suspension of 150 µL of Sera-Mag Magnetic Streptavidin Coated Particles was used (high binding capacity: 4500 to 5500 pmol/mg, GE Healthcare, Chicago, IL, USA). Each time the beads were collected for 5 min in a 16-Tube SureBeads Magnetic Rack (Bio-Rad, Hercules, CA, USA). Initially the beads were washed three times in 1 mL of PBS by resuspension. Incubations described further were carried out at 4 °C with gentle rocking. A solution of DNA (20 μg) in 1 mL of PBS was added to the washed beads and incubated for 1 h. Next, the beads were incubated for 15 min in 1 mL of PBS with biotin (Sigma Aldrich, Darmstadt, Germany) at the final concentration of 800 μg/mL. Subsequently, the beads were incubated for 1 h with 2 mL of *S. aureus* whole-cell lysate supplemented with biotin and poli (dI:dC) (Sigma Aldrich, Darmstadt, Germany) at a final concentration of 10 μg/mL and 2 μg/mL, respectively, and washed three times with 1 mL of lysing buffer that was used for whole-cell lysate preparation. Finally, the beads were resuspended in 100 μL of SDS-PAGE loading buffer (60 mM Tris; 2% SDS; 10% *v/v* glycerol; 0.025% CBB G-250; 100 mM DTT; pH 8.45), incubated at 95 °C for 5 min, and collected. The aspirated supernatant was stored at −20 °C. Subsequently, 25 μL of supernatant samples was used for SDS-PAGE analysis, which was carried out in the Laemmli system with Coomasie Brilliant Blue R-250 used for gel staining [62]. Unstained Protein MW Marker (Thermo Scientific, Waltham, MA, USA) was used as a mass standard.

**Protein isolation from acrylamide gel and mass spectrometry**. The procedure used for protein isolation from Coomasie-stained polyacrylamide gel and trypsin digestion for mass spectrometry was adapted from Shevchenko et al. [63]. Subsequent steps of the procedure were carried out at room temperature if not otherwise specified. Once SDS-PAGE was finished, Coomasie-stained gels were washed in distilled water for 10 min twice. The selected bands were excised, cut into small fragments, and transferred to a 1.5 mL tube. Gel fragments were washed in 50 μL of wash buffer (10 mM NH_4_HCO_3_ with 50% acetonitrile in water) for 15 min. Next, the liquid was aspirated, and gel fragments incubated in 100 μL of acetonitrile until they shrunk. After that, acetonitrile removal gel fragments were rehydrated in 25 μL 20 mM NH_4_HCO_3_ for 5 min. Next, gel fragments were mixed with 25 μL of acetonitrile and incubated for 15 min. After the liquid was aspirated, 100 μL of acetonitrile was added, and gel fragments incubated for 5 min. The liquid was removed, and gel fragments air-dried. To perform reduction and subsequent alkylation, 50 μL of a freshly prepared solution of 10 mM DTT with 10 mM NH_4_HCO_3_ was added to dry gel fragments, and the mixture was incubated for 15 min at 56 °C. After cooling the samples back down to room temperature, 50 μL of a freshly prepared solution of 55 mM iodoacetamide with 10 mM NH_4_HCO_3_ was added to gel fragments and incubated for 20 min in the dark. After liquid removal, the gel fragments were washed with 50 μL of wash buffer for 15 min and incubated with 100 μL of acetonitrile until they shrunk. After acetonitrile removal, gel fragments were air-dried. Next in-gel digestion was performed. Dry gel fragments were mixed with 3 μL of Trypsin Gold Mass Spectrometry Grade (Promega, Madison, WI, USA, 20 ng/μL; 10 mM NH_4_HCO_3_) and incubated at 4 °C for 60 min. Subsequently, 10 μL of 10 mM NH_4_HCO_3_ was added and the incubation continued at 37 °C overnight. In the last stage, peptide extraction was carried out. A volume of 5 μL of 50% acetonitrile with 1% trifluoroacetic acid in water was added to the digestion mixture. Samples were subsequently incubated for 45 min in an Elmasonic S15H ultrasonic bath (Elma, Lomza, Poland) at room temperature. Mass spectrometry identification of peptides present in the aspirated liquid was carried out in the same setting as in Bonar et al. [64].

**Total RNA isolation**. Samples of around 4.0/OD_600_ mL in the case of RT-qPCR or of 5 mL in the case of RNA-Seq were collected from analysed cultures to 2 or 5 mL tubes and centrifuged at 5000 RCF at room temperature for 2 or 5 min, respectively. After supernatant aspiration bacteria from each sample were suspended in 1 mL of Tri-Reagent (Bioshop, ON, Canada), transferred to 2 mL Lysing Matrix B tubes (MP Biomedicals, Illkirch-Graffenstaden, France) and subjected to mechanical disruption in a Precellys device (Bertin Instruments, Montigny-le-Bretonneux, France) according to the following programme: 4500 RPM speed for 10 cycles, 90 s each with 1 s break. Next, 100 μL of 1-bromo-3-chloropropane (Sigma Aldrich, Darmstadt, Germany) was added and the samples were vigorously shaken by hand for 20 s and left at room temperature for 5 min to let the phases separate. Next, the samples were centrifuged at 12,000 RCF at 4 °C for 15 min. A volume of 400 μL of the top-most water phase was transferred to fresh tubes and supplemented with 200 μL of 96% ethanol. Further purification steps were carried out using GeneJET RNA Purification Kit (Thermo Scientific, Waltham, MA, USA) in accordance with the manufacturer’s recommendations. After binding and before the washing step, DNA contamination was removed using On-Column DNase I Digestion Set (Sigma Aldrich, Dramstadt, Germany) in accordance with the manufacturer’s protocol. RNA was eluted in 50 μL of double-distilled RNAse/DNAse-free water (ddH_2_O, EURx, Gdansk, Poland). The integrity of RNA was assessed by agarose gel electrophoresis in denaturing conditions [65]. In the case of RNA-Seq analysis, obtained RNA samples were additionally quantified using Qubit 4.0 fluorometer (Invitrogen, Waltham, MA, USA) and its quality was assessed with RNA 6000 Pico Kit and Prokaryote Total assay using 2100 Bioanalyzer System (Agilent Technologies, Santa Clara, CA, USA). Only samples with preserved 16S and 23S peaks and RIN values greater than 9 were selected for further analysis. Samples were stored at −80 °C.

**Gene transcription analysis with RT-qPCR**. Reverse transcription was performed using M-MuLV RevertAid Premium Reverse Transcriptase (Thermo Scientific, Waltham, MA, USA) and random hexamers (Genomed, Warsaw, Poland) in accordance with the manufacturer’s recommendations. For each reaction 2 μg of purified RNA were used. Upon completion, reaction mixtures with cDNA were diluted 10-fold with ddH_2_O. The length of fragments amplified during qPCR fell within a range of 60–80 bp. Premix solutions were prepared, consisting of 22 μL of ddH_2_O, 33 μL of a pair of gene-specific primers in ddH_2_O, 10 μM each, and 165 μL iTaq Universal Sybr Green Super Mix (Bio-Rad, Hercules, CA, USA). To a 96-well plate, 5 μL portions of cDNA were aliquoted in triplicate. Next, 10 μL portions of premixes were added to each well. The plate was sealed with adhesive foil and centrifuged for 5 min at 1000 RFC. The qPCR reactions were carried out in CFX96 Touch™ Real-Time PCR Detection System (Bio-Rad, Hercules, CA, USA) according to the following programme: 1. 95 °C 2 min; 2. 95 °C 30 s; 3. 55 °C 30 s (measurement); 4. 74 °C 30 s; 5. repeat steps 2, 3, 4 for 40 cycles; measure melting curves in the range from 65 to 95 °C with an increment of 0.5 °C. The relative expression of the genes of *saoABC* operon in reference to *gryB* and 23S rRNA transcripts were calculated with the ∆∆Ct method according to Livak and Schmittgena [66] independently for each replicate. Statistical significance of differences between control and experimental cultures at subsequent time points was estimated using a *t*-test.

**RNA-Seq transcriptomics**. Bacteria were grown as described in the section on growth of control cultures in stress conditions in TSB in three independent biological replicates. Samples were collected at 20 min (logarithmic growth phase) and 4 h (late growth phase) after medium change. Total RNA isolated from cultures was depleted of rRNA using MICROBExpress Bacterial mRNA Enrichment Kit (Thermo Scientific, Waltham, MA, USA) with a total amount of 5 µg of input RNA, and subsequently ethanol precipitation was applied for 1 h at −20 °C and finally RNA was resuspended in 25 µL of ddH_2_O. The effectiveness of rRNA depletion was validated with Bioanalyzer System (Agilent Technologies, Santa Clara, CA, USA) using RNA 6000 Pico Kit and mRNA assay. Libraries were prepared using TruSeq Stranded mRNA kit (Illumina, San Diego, CA, USA) in accordance with the manufacturer’s instructions. Briefly, the purified mRNA was quantified using Qubit RNA HS assay kit and Qubit 4.0 Fluorimeter (Invitrogen, Waltham, MA, USA) and diluted to 20 ng/µL. The first and second strand of cDNA were synthesised and then purified using Agencourt AMPure XP beads (Beckman Coulter, Brea, CA, USA). Next, adenylation of 3′ ends and ligation of multiple indexing adapters to the ends of the double-stranded cDNA followed by AMPure XP beads purification were performed. Finally, cDNA fragments were enriched by PCR and again purified with AMPure XP beads. The length distribution of obtained fragments was estimated with 2100 Bioanalyzer System and Agilent High Sensitivity DNA kit (Agilent Technologies, Santa Clara, CA, USA), in accordance with the manufacturer’s recommendations. The length of obtained fragments fell between 200 and 300 bp. The indexed libraries were normalised, pooled, and loaded onto an Illumina MiSeq reagent cartridge with MiSeq reagent kit v3 (Illumina, San Diego, CA, USA) for 150 cycles. The paired-end 2 × 75  bp sequencing was run on Illumina MiSeq sequencer resulting in over 20 million reads PF, Q30 ranging from 95.62 to 97.71%, and cluster density from 1075 to 1445. The reads are available at the NCBI SRA database through BioProject accession number PRJNA798259.

**Differential gene expression calculation**. Quantification of gene expression based on RNA-Seq raw reads was carried out using Salmon (ver. 1.4.0) [67]. Reference sequences were obtained from *S. aureus* NCTC 8325 complete genome sequence (GenBank CP000253.1) [68]. Those were sequences annotated as CDS and tRNA as well as sequences of small RNAs as indicated in the SRD database [69]. In the case of duplicated annotations, annotations of exact gene duplicates, or annotations located on the same strand that were overlapping each other to a substantial extent, only one annotation was analysed. The multiple sequence FASTA file used to create the index is provided as supplementary file NCTC_8325_RNA_Seq.fna. For index creation, k-mer length of 15 was used. Quantification was carried out with indication of the library type as paired-read inward stranded reverse (ISR) for gene expression analysis, and paired-read inward stranded forward (ISF) for anti-sense RNA expression analysis. Differential gene expression (DGE) was assessed using Bioconductor library DESeq2 (ver. 1.30.0) [70]. The complete analysis was facilitated by in-house Python (ver. 3.8.5) and R (ver. 4.0.3) scripts combined together with Salmon in one Snakemake (ver. 5.32.0) pipeline [71]. All of the script files were uploaded to GitHub and are available at https://github.com/michalbukowski/rnaseq_pipeline, together with a link to a complete environment set-up delivered as a Docker image. Raw output data on DGE is provided as supplementary spreadsheet file DGE.xlsx. The ontology analysis was performed using in-house Python scripts in Jupyter (ver. 4.7.1) notebooks and based on metadata on the *S. aureus* NCTC 8325 genome available at the AureoWiki repository [72] enriched with annotations based on COG ontologies [73], virulence factor database VFDB [74] and the staphylococcal regulatory RNA database SDR [69]. The combined metadata on the *S. aureus* NCTC 8325 genome that have been used are provided in supplementary spreadsheet file NCTC_8325_metadata.xlsx.

**Persister formation analysis**. Bacteria were grown overnight in 20 mL MHB medium (Sigma-Aldrich, Darmstadt, Germany) at 37 °C with 180 RPM shaking. Fresh, pre-warmed 20 mL portions of MHB medium were inoculated with 20 μL of the overnight cultures and incubated at the same conditions until cultures reached OD_600_ equal to 0.1. In the case of strains transformed with pCN51-saoB or pCN51-saoC plasmid constructs, culture medium was supplemented with erythromycin at the final concentration of 10 μg/mL, and the expression of genes was induced with CdCl_2_ at a final concentration of 2.5 μM, for 1 h. Next, samples of 0.1/OD_600_ × 200 μL in volume were transferred to fresh, prewarmed 20 mL portions of MHB medium supplemented with penicillin at the final concentration of 1 μg/mL, and, when desirable, erythromycin at the final concentration of 10 μg/mL. Cultures were kept at the initial growth conditions for 4 h. Subsequently, 1 mL samples were collected at the start and at the end time point for these cultures and centrifuged for 2 min at 8000 RFC. The samples for the start time point were collected prior to penicillin supplementation. After supernatant was discarded, bacteria were resuspended in 1 mL PBS and additionally 10-fold diluted prior to being plated on 90 mm Petri dishes with TSA medium (Sigma-Aldrich, Darmstadt, Germany) with an Eddy Jet Spiral plater (IUL, Barcelona, Spain) using the Log50 programme. After overnight incubation at 37 °C, colony counting was carried out in accordance with the device manual, and the persister fractions were calculated for the endpoint sample in respect to the sample from the starting time point. Statistical significance of the observed differences was assessed using two-tailed Mann–Whitney U test with p-value threshold at 0.05.

**Intracellular survival determination**. A volume of 150 μL of 0.1% gelatine (Sigma-Aldrich, Darmstadt, Germany) solution in PBS (Dulbecco’s Phosphate Buffered Saline without calcium and magnesium, Biowest, Nuaille, France) was aliquoted to a 24-well plate. The plate was subsequently incubated for 1 h at 37 °C. The gelatine solution was aspirated, the wells washed tree times with 0.5 mL of PBS, and finally filled with 0.5 mL DMEM medium (high glucose with L-glutamine and sodium pyruvate, Biowest, Nuaille, France) supplemented with 10% FBS (fetal bovine serum, South America, Biowest, Nuaille, France) and antibiotics (Penicillin-Streptomycin Solution 100× and Amphotericin 100× solutions, Biowest, Nuaille, France). To each well, 50,000 cells of human dermal fibroblasts (HDF, Sigma-Aldrich, Darmstadt, Germany) were added and grown until they reached confluence, which was 400,000 cells per well. Bacterial strains were grown as described before in TSA medium additionally supplemented with 10% skimmed milk. Fresh cultures were prepared by 1:100 dilution of overnight ones and grown in the same conditions until OD_600_ reached 2.5. Bacteria were washed with PBS prior to the density measurement. Final samples were washed three times, suspended in PBS, and OD_600_ was determined yet again. The volume of 1/OD_600_ × 300 μL was added to 15 mL of DMEM supplemented with 4% FBS to achieve a suspension of 2 × 10^7^ CFU (colony forming units).

From each well with fibroblasts, medium was aspirated, and the cells were washed with PBS. A volume of 1 mL of each of analysed bacterial suspension was added in triplicate to the wells to achieve multiplicity of infection (MOI) equal to 50. The plate was incubated for 45 min at 37 °C, and subsequently the wells were washed three times with PBS and filled with 1 mL of DMEM supplemented with 4% FBS and gentamycin at the final concentration of 100 μg/mL (Bioshop, ON, Canada). The first measurement was carried out after an additional 30 min of incubation, which was 1.5 h after inoculation with bacteria. Next, time points were set to 3 and 6 h. The wells were washed with PBS and to each well 1 mL of ddH_2_O was added. The plate was then incubated at 37 °C for 15 min. After that, cells were scratched off the bottom of wells, the suspensions transferred to 1.5 mL tubes and treated with 10 pulses from a Hielscher UP50H sonicator at cycle length of 0.5 and amplitude of 80%. Samples of these suspensions were plated on 90 mm Petri dishes with TSA in an Eddy Jet Spiral Plater (IUL, Barcelona, Spain) using the C-mode 40 programme. The plates were incubated overnight at 37 °C, and colonies were counted to determine CFU in analysed suspensions.

## Figures and Tables

**Table 1:** Transcripts differentially expressed in RN4220 Δ*saoC* mutant in the logarithmic growth phase. Transcripts, where applicable, are grouped into operons (capital letters in subscripts of locus tags) with the genomic order preserved, and then sorted in respect to the fold change value. In the case of operons, locus with the largest absolute fold change was taken into account whilst sorting. Negative values indicate fold-decrease in transcription. FDR, false discovery rate (*p* value adjusted with Benjamini and Hochberg method). Ontologies are determined based on the transcript type (non-coding RNA), COG database (COG IDs given in parentheses), Virulence Factor Database (VFDBIDs given in parentheses) or available literature (indicated).

Locus Tag/Operon	Gene	Fold Change	FDR	Product	Ontology
srn_9200_sRNA101		−49.94	1.04 × 10^−2^	Non-coding RNA srn_9200_sRNA101	Non-coding RNA of unknown function
SAOUHSC_02243_A_	* lukH *	−2.98	3.74 × 10^−5^	Beta-channel forming cytolysin, aerolysin/leukocidin family protein	Virulence factors (TX425)
SAOUHSC_02241_A_	* lukG *	−2.69	4.53 × 10^−6^	Beta-channel forming cytolysin, leukocidin/hemolysin toxin family protein	Virulence factors (TX425)
SAOUHSC_00399	* ssl11 *	−2.96	4.96 × 10^−2^	Superantigen-like protein, exotoxin	Virulence factors (CVF075)
SAOUHSC_00010_B_		−2.94	5.21 × 10^−4^	Predicted branched-chain amino acid permease (azaleucine resistance)	Amino acid transport and metabolism (COG1296)
SAOUHSC_00012_B_		−2.50	4.49 × 10^−2^	Branched-chain amino acid transport protein	Amino acid transport and metabolism (COG4392)
SAOUHSC_00899_C_	* argG *	−2.37	1.86 × 10^−4^	Argininosuccinate synthase	Amino acid transport and metabolism (COG0137)
SAOUHSC_00898_C_	* argH *	−2.56	5.05 × 10^−3^	Argininosuccinate lyase	Amino acid transport and metabolism (COG0165)
SAOUHSC_01991 _ D _		−2.34	9.00 × 10^−8^	ABC transporter permease	Amino acid transport and metabolism (COG0765)
SAOUHSC_01990_D_	* glnQ *	−2.48	4.62 × 10^−4^	Amino acid ABC transporter ATP-binding protein	Amino acid transport and metabolism (COG1126)
srn_4140_sRNA338		−2.39	1.94 × 10^−2^	Non-coding RNA srn_4140_sRNA338	Non-coding RNA
SAOUHSC_00400		−2.29	9.20 × 10^−5^	Gram-positive signal peptide, YSIRK family	
SAOUHSC_02967_E_	* arcD *	−2.13	1.97 × 10^−4^	Arginine-ornithine antiporter	Amino acid transport and metabolism (COG0531)
SAOUHSC_02968_E_	* arcB *	−2.04	1.90 × 10^−3^	Ornithine carbamoyltransferase	Amino acid transport and metabolism (COG0078)
SAOUHSC_01363		2.05	7.08 × 10^−3^	Nucleotidyltransferase/DNA polymerase involved in DNA repair	Replication, recombination and repair (COG0389)
srn_3270_sRNA259		2.13	3.87 × 10^−2^	Non-coding RNA srn_3270_sRNA259	Non-coding RNA
SAOUHSC_03006	* gehA *	2.22	1.18 × 10^−2^	Triacylglycerol lipase precursor	Lipid transport and metabolism (COG1075) and Virulence factors (CVF091)
SAOUHSC_00912	* clpB *	2.26	6.14 × 10^−27^	ATP-dependent Clp protease ATP-binding, subunit ClpB	Posttranslational modification, protein turnover, chaperones (COG0542)
SAOUHSC_00013	* metX *	2.28	5.50 × 10^−3^	Homoserine O-acetyltransferase	Amino acid transport and metabolism (COG2021)
srn_3780_Teg13		2.30	6.93 × 10^−4^	Non-coding RNA srn_3780_Teg13	Non-coding RNA
SAOUHSC_00561	* vraX *	2.32	8.37 × 10^−4^	Protein VraX	Virulence factors [34]
SAOUHSC_00435	* gltB *	2.40	1.73 × 10^−7^	Glutamate synthase, large subunit	Amino acid transport and metabolism (COG0069)
SAOUHSC_01334		2.60	1.94 × 10^−2^	Hypothetical protein	
SAOUHSC_00369	* saoC *	2.63	6.13 × 10^−96^	Stress associated protein SaoC	
SAOUHSC_00174		2.88	3.39 × 10^−2^	Murein DD-endopeptidase MepM and murein hydrolase activator NlpD, contain LysM domain	Cell wall, membrane, envelope biogenesis (COG0739)
srn_1490_Sau6477		3.16	2.29 × 10^−19^	Non-coding RNA srn_1490_Sau6477	Non-coding RNA
SAOUHSC_03025	* pcp *	3.17	2.92 × 10^−5^	Pyrrolidone-carboxylate peptidase (N-terminal pyroglutamyl peptidase)	Posttranslational modification, protein turnover, chaperones (COG2039)
SAOUHSC_00347		3.47	2.39 × 10^−4^	Hypothetical protein	
SAOUHSC_02144		3.84	1.94 × 10^−6^	Hypothetical protein	
SAOUHSC_00556 antisense RNA	* proP *	5.53	9.28 × 10^−3^	Predicted arabinose efflux permease, MFS family	Carbohydrate transport and metabolism (COG2814)

**Table 2:** Transcripts differentially expressed in RN4220 Δ*saoC* mutant in the late growth phase. Transcripts are sorted in respect to the fold change value. Negative values indicate fold-decrease in transcription. FDR, false discovery rate (*p* value adjusted with Benjamini and Hochberg method). Ontologies are determined based on the transcript type (non-coding RNA, transfer RNA) and COG database (COG IDs given in parentheses).

Locus Tag	Gene	Fold Change	FDR	Product	Ontology
SAOUHSC_T00052	* trnaS *	−58.16	4.32 × 10^−3^	tRNA-Ser	Transfer RNA
SAOUHSC_02741	* opuCD *	−2.03	3.01 × 10^−2^	ABC-type proline/glycine betaine transport system, permease component	Amino acid transport and metabolism (COG1174)
SAOUHSC_02108	* ftnA *	2.47	1.81 × 10^−2^	Ferritin	Inorganic ion transport and metabolism (COG1528)
srn_4140_sRNA338		2.73	8.06 × 10^−3^	Non-coding RNA srn_4140_sRNA338	Non-coding RNA
SAOUHSC_02423		2.76	1.92 × 10^−2^	UDP-N-acetylglucosamine pyrophosphorylase	Carbohydrate transport and metabolism (COG4284)
SAOUHSC_00369	* saoC *	10.98	2.89 × 10^−78^	Stress associated protein SaoC	

## Data Availability

RNA-Seq reads are available at the NCBI SRA database through BioProject accession number PRJNA798259. The complete pipeline, which was used for RNA-Seq data analysis, including in-house Python and R scripts and a link to a complete environment set-up in a Docker image, is available at https://github.com/michalbukowski/rnaseq_pipeline.

## Supplementary Materials

The following supporting information can be downloaded at: https://www.mdpi.com/article/10.3390/ijms23126443/s1.
