# Supplementary material for: Staphylococcal saoABC Operon Codes for a DNA-Binding Protein SaoC Implicated in the Response to Nutrient Deficit
Source: Int J Mol Sci. 2022 Jun 9;23(12):6443. doi: 10.3390/ijms23126443 (PMC9223772; doi:10.3390/ijms23126443)
Supplement: Supplementary file 1 [file ijms-23-06443-s001.zip › saoABC_suppl_tables_and_figures.zip!/saoABC_suppl_tables_and_figures.pdf]

Staphylococcal *saoABC* operon codes for a DNA-binding protein SaoC implicated in the response to nutrient deficit

Michal Bukowski [1,*], Maja Kosecka-Strojek [2], Anna Madry [1], Rafal Zagorski-Przybylo [1], Tomasz Zadlo [1], Katarzyna Gawron [3], Benedykt Wladyka [1]

[1] Department of Analytical Biochemistry, Faculty of Biochemistry, Biophysics and Biotechnology, Jagiellonian University in Krakow, Krakow, Poland
[2] Department of Microbiology, Faculty of Biochemistry, Biophysics and Biotechnology, Jagiellonian University in Krakow, Krakow, Poland
[3] Department of Molecular Biology and Genetics, Faculty of Medical Sciences in Katowice, Medical University of Silesia, Katowice, Poland
* Correspondence: m.bukowski@uj.edu.pl

List S1. Occurrence of *saoABC* operon among staphylococcal species. The *saoABC* operon has been found in 47 out of 51 staphylococcal species. Negative species are underlined. It should be noted that in case of 4 species, in genomes of which we were unable to find the operon, the number of available genomic sequences was low (<10). Results for these species might be thus unreliable and in the future *saoABC* operon will likely be found in those species as well.

1. *S. agnetis*
2. *S. argensis*
3. *S. argenteus*
4. *S. arlettae*
5. *S. aureus*
6. *S. auricularis*
7. *S. capitis*
8. *S. caprae*
9. *S. carnosus*
10. *S. chromogenes*
11. *S. cohnii*
12. *S. condimenti*
13. *S. cornubiensis*
14. *S. delphini*
15. *S. devriesei*
16. *S. edaphicus*
17. *S. epidermidis*
18. *S. equorum*
19. *S. felis*
20. *S. fleurettii*
21. *S. gallinarum*
22. *S. haemolyticus*
23. *S. hominis*
24. *S. hyicus*
25. *S. intermedius*
26. *S. kloosii*
27. *S. lentus*
28. *S. lugdunensis*
29. *S. lutrae*
30. *S. massiliensis*
31. *S. microti*
32. *S. muscae*
33. *S. nepalensis*
34. *S. pasteuri*
35. *S. petrasii*
36. *S. pettenkoferi*
37. *S. piscifermentans*
38. *S. pseudintermedius*
39. *S. rostri*
40. *S. saccharolyticus*
41. *S. saprophyticus*
42. *S. schleiferi*
43. *S. schweitzeri*
44. *S. sciuri*
45. *S. simiae*
46. *S. simulans*
47. *S. stepanovicii*
48. *S. succinus*
49. *S. vitulinus*
50. *S. warneri*
51. *S. xylosus*

Table S1. Primers used in the study. Restriction sites are double-underlined, start and stop codons as well as recombinase attachment sites (*attB1*/*attB2*) are in bold, ribosome-binding sites (RBSs) are underlined, group II Ll.LtrB-ΔORF intron target sequences are in bold and underlined.

Name	Sequence	Use
pD-saoC-F	ATACAT**ATG**TTAACTAAAGAATTTGCACAACG	pETDuet-saoC (NdeI/XhoI)
pD-saoC-R	AGACTCGAG**TTA**CAGGTTGAATAAACG	
pC-saoB-F	CCGGTCGAC**TAACTAACTAAT**GGGGAGGTGTTCTT**ATG**G	pCN51-saoB (SalI/BamHI)
pC-saoB-R	CGCGGATCC**TTA**AAATGGTTTACGTAAATCCATTTC	
pC-saoC-F	CCGGTCGAC**TAACTAACTAAT**GCAGAAAGGACTTTAAAGT**ATG**	pCN51-saoC (SalI/BamHI)
pC-saoC-F	CGCGGATCC**TTA**CAGGTTGAATAAACGTGCG	
pK-saoA-attB1-UF	**GGGGACAAGTTTGTACAAAAAAGCAGGCT**TGATGCCCATTTTGGAAACTCTTTTC	pKOR-del-saoA (*attB1*/SacII/*attB2*)
pK-saoA-UR	AGGTCCCCGCGGAGTTGTCGCCCCTTTAAAAATTTG	
pK-saoA-DF	AGGTCCCCGCGGTTATCACACTGAATTCAAAATGAGATTAC	
pK-saoA-attB2-DR	**GGGGACCACTTTGTACAAGAAAGCTGGGT**TTCTTCTAAATGTTGAACAATTTTACGGAC	
pK-saoC-attB1-F	**GGGGACAAGTTTGTACAAAAAAGCAGGCT**GTTCAAGAACATAACTCAGCATATG	pKOR-del-saoC (*attB1*/SacII/*attB2*)
pK-saoC-UR	AGGTCCCCGCGGACTTTAAAGTCCTTTCTGCATATAC	
pK-saoC-DF	AGGTCCCCGCGGACTTGTGATTTGAACACAAATTTAATAC	
pK-saoC-attB2-R	**GGGGACCACTTTGTACAAGAAAGCTGGGT**ATCAGTGTAAGAAAAGCATTCATGAAC	
pN-IBS1-saoB	AAAAAAGCTTATAATTATCCTTA**TATTACCAAACC**GTGCGCCCAGATAGGGTG	pNL9164-saoB
pN-EBS1-saoB	CAGATTGTACAAATGTGGTGATAACAGATAAGTC**CAAACCTT**TAACTTACCTTTCTTTGT	
pN-EBS2-saoB	TGAACGCAAGTTTCTAATTTCGGTT**TAATA**TCGATAGAGGAAAGTGTCT	
pN-IBS1-saoC	AAAAAAGCTTATAATTATCCTTA**GTAGACGATTTA**GTGCGCCCAGATAGGGTG	pNL9164-saoC
pN-EBS1-saoC	CAGATTGTACAAATGTGGTGATAACAGATAAGTC**GATTTACG**TAACTTACCTTTCTTTGT	
pN-EBS2-saoC	TGAACGCAAGTTTCTAATTTCGATT**TCTAC**TCGATAGAGGAAAGTGTCT	

pC-P-saoABC-F	GCTGCATGCATAACTGCTTAGGTAATCTT	pCN35-P-saoABC-gfpmut2-term
pC-P-saoABC-R	AGAGTCGACCAGGTTGAATAAACGTG	(PaeI/SalI/BamHI/EcoRI)
pC-gfpmut2-F	CAGGTCGACAGTAAAGGAGAAGAACTTTTC	
pC-gfpmut2-R	CGGGGATCC**TTA**TTTGTATAGTTCATCCATG	
pC-term-F	AGAGGATCCACTTGTGATTTGAACACAAATTT	
pC-term-R	CCTGAATTCAGGAATACACAGGAGCCA	
RT-saoA-F	TGTTAGTTGTAGCGGTTCGTG	RT-qPCR
RT-saoA-R	GCTTTGGGACCTTTAGATGCT	
RT-saoB-F	AGATTTAACACAAGATAACGGCCTC	
RT-saoB-R	GTGTGACCATCATTAGTTGGATGAA	
RT-saoC-F	ATCAACAGACGCTAAGACGGA	
RT-saoC-R	GCTCAACGCTGGCTTTTTCA	
RT-gyrB-F	CAACAATGAACCCTGAGCACC	
RT-gyrB-R	CGGTTTTCTACAACGTCACCC	
RT-23SRNA-F	CCGAAGCTGTGGATTGTCCT	
RT-23SRNA-R	GCTACTCACACCGGCATTCT	
saoA-prom-F	AAGTTTCATCAAATAAACTGTGACCATC	DNA pull-down
saoA-prom-R	TGTTCACCATCTTCTCCTGTTAAC	
saoC-prom-F	TTCATCCAACTAATGATGGTCACAC	
saoC-prom-R	TCTAATTCATCAAACGCTAGATCATAAC	
saoC-prom-fr1.2-F	ATTTATGCTTTATTAGAAGAAATGGATTTACG	
saoC-prom-fr1-R	TGAAACGTCAGTCTTGCTTCATAC	
saoC-prom-fr2-R	TATACTGACGTTTGTCTACGGG	
saoC-prom-fr3-F	TTTAAACTATGTATGAAGCAAGACTGAC	
saoC-prom-fr3-R	CGTTGTGCAAATTCTTTAGTTAACATAC	
saoC-rand-prom-F	CAAATAATAGGTACAAATCTAAAGACGG	Control for DNA pull-down
saoC-rand-prom-R1	CTCTCACTATTTTTATACGCCTTTTTATG	
saoC-rand-prom-R2	TGACTATAAGTAGCTAAAACATAATGTATTTG	

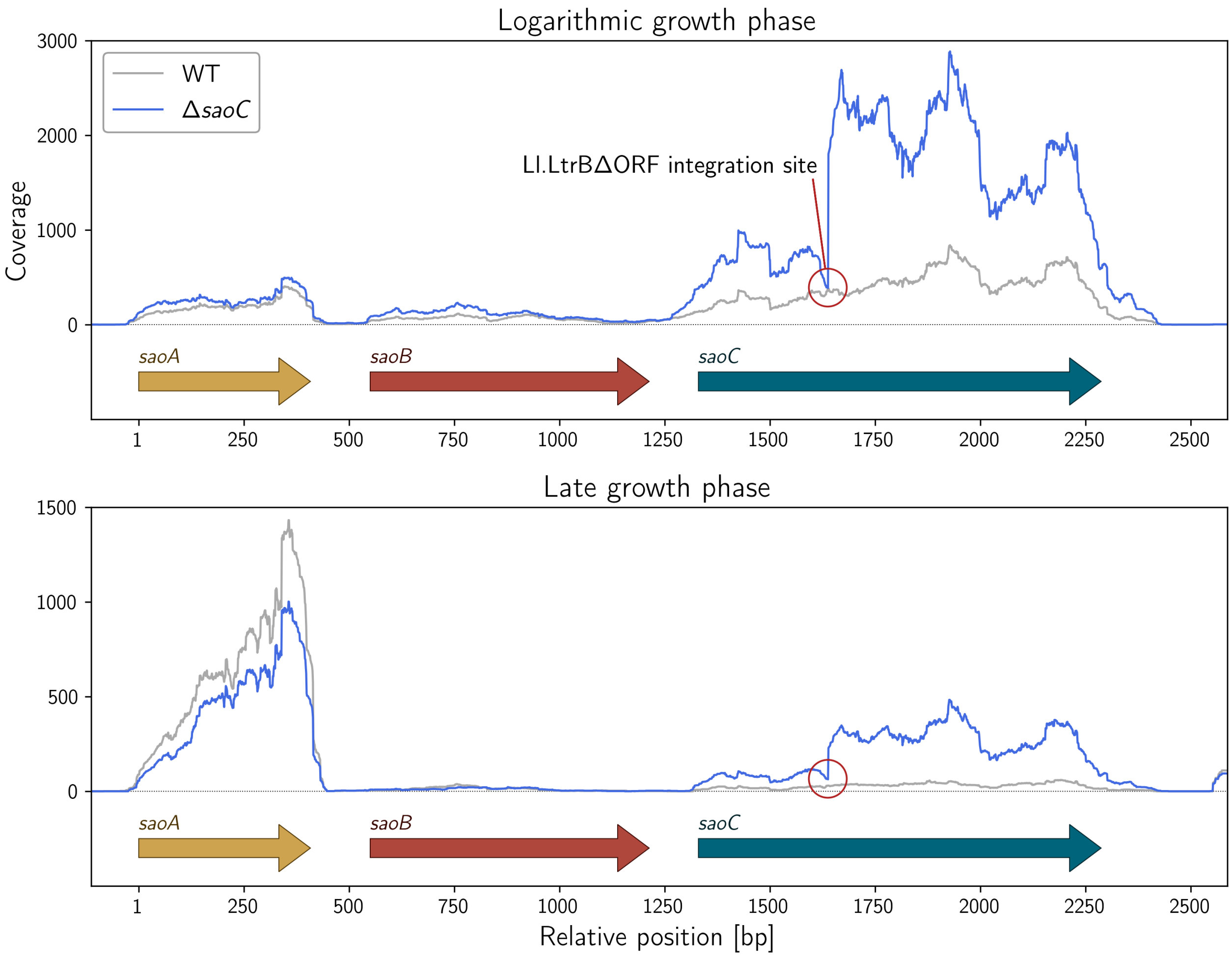

Figure S1. Coverage plots of reads mapped specifically to *saoABC* operon. The reads were derived from all RNA-Seq replicas either for the logarithmic (upper plot) or the late phase of growth (lower plot). In both cases clearly elevated coverage of *saoC* (disrupted by Ll.LtrBΔORF intron) might be observed for Δ*saoC* mutant, which corroborate the claim that functional SaoC acts as a transcriptional repressor by binding to the conserved regulatory sequence upstream its own gene.

```
MbtS    1 ---------MNLS-KQIKK---YRERDGYSQEYLAEKLYVSRQS------   31
                   :.|| ||::|   :.|..||.   |::..|..|::
SaoC    1 MLTKEFAQRVELSEKQVRKIVQHLEERGYQ---LSKTEYRGREATDFKEE   47

MbtS   32 -----------------------------------ISNWENDKSLPDIHN   46
                                             :.|  :||:||...|
SaoC   48 DIELFKDIADKVKQTNSYDLAFDELEKEKDFLQVIVKN--DDKNLPTNQN   95

MbtS   47 LLMMCELFNVTLDDLVKGTIPFVPDIKAQRSLNLWTYVMLIFMTLAAILM   96
          :..:
SaoC   96 VAQL----------------------------------------------   99

MbtS   97 GPLVVYWNWTWGVTVAIILGIGFYASMKIEDLK-----------------  129
                                      :|||:
SaoC  100 ----------------------------VEDLRLEIQKMREERHLLGQMM  121

MbtS  130 -KVH----------------------------------------------  132
           :||
SaoC  122 NQVHQQQQELKELQNQLTSKIDSNSESLKAIQTSQEAIQEAQASQAKVLA  171

MbtS  133 --------------KMDNYDRIVAFMNGKDPSEVQT--------------  154
                        |.|:.|..||.:|....::..|
SaoC  172 ESTNKVEKNAVTEDKADSKDSKVAGVNTSTDAKTDTKADNAGDGTTTKVD  221

MbtS  154 --------------------------------------------------  154

SaoC  222 KEDQISATEAIEKASVEQSNGNAAETSNKEATVDAEAQHDAEQQVAEAHA  271

MbtS  155 -------------TKARNTMTNALSIIS--------------VIGILSLI  177
                       .||.|..|.:.|.:|              :..:
SaoC  272 EASKQATSNDSLEAKAENDSTASQSEMSEPKPQEEKKGFFARLFNL----  317

MbtS  178 IFLSVYLANKFL  189

SaoC  317 ------------  317
```

Figure S2. Sequence alignment of MbtS, a transcription factor recently described for *S. aureus* [1], and SaoC. Sequence similarity between these two proteins is limited.

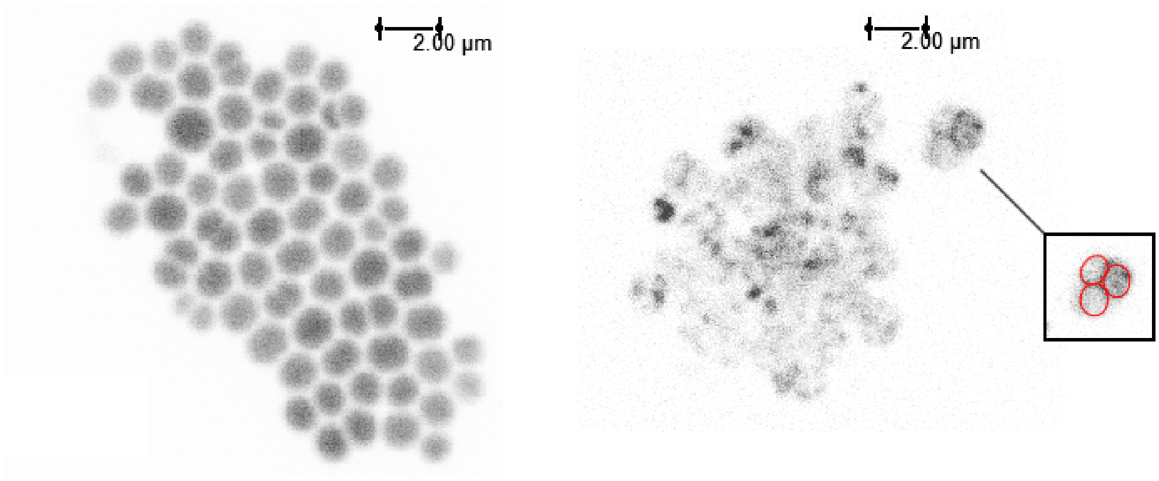

Figure S3. Confocal microscopy picture of *S. aureus* RN4220 transformed with pCN68 for free GFP expression (left) and pCN35-P-saoABC-gfpmut2-term carrying a copy of complete *saoABC* operon with *gfpmut-2* sequence insert for expression of SaoC-GFP fusion protein (right). In the latter case fluorescence signal forms ring-shaped halos, which indicates peripheral localisation of SaoC-GFP, likely at the inner surface of cell membrane.

CAAATAATAGGTACAAATCTAAAGACGGTAAGTGCTAGCCAACCTTGTATGATGCATTGT
AATAACTTCGTAAATAATAATAATTAACAAATACATTATGTTTTAGCTACTTATAGTCAC
CATAGCAAATAGTCGCTTCTAAGATAATCCACCAAATTTATTACTTATAGGTCACATTGT
AAGCTAGTCCGAGGAGTTTAAAAAGAGTGTTGCCATGATTTAGGGATTTGTAACCAGTCA
AAGGTCAACTTTCTTATATGGATTTGAACAAGAAATAGTGTTTTCTAGTTTTCTTCAATA
CGTCATAGTTGATGTGCTATCTCCAATTCACAGTATCTATTTCTGAATTGGACATGGCTG
AAGAAGGGAAAAGTTGAGGGTAGTCACAAAGTTTAAAGAATATTACATGGAATCGCCAGA
ATGAACTAAAATCATAGGCTTCCTTATGAAGTATCCCTCAGCGAACTGTCACACGGTAAC
TAAAGAGAAGAATGATCATAAAAAGGCGTATAAAAATAGTGAGAGT

Sequence S1. Shuffled *saoC* promoter sequence (saoC-rand-prom) used as a control for DNA pull-down experiments. Hybridisation sites for primers are underlined.

References

1. Yeo, W.S.; Anokwute, C.; Marcadis, P.; Levitan, M.; Ahmed, M.; Bae, Y.; Kim, K.; Kostrominova, T.; Liu, Q.; Bae, T. A Membrane-Bound Transcription Factor Is Proteolytically Regulated by the Aaa+ Protease Ftsh in Staphylococcus Aureus. *J. Bacteriol.* **2020**, *202*, e00019-20. https://doi.org/10.1128/JB.00019-20.
